# Blood-based CNS regionally and neuronally enriched extracellular vesicles carrying pTau217 for Alzheimer’s disease diagnosis and differential diagnosis

**DOI:** 10.1186/s40478-024-01727-w

**Published:** 2024-03-05

**Authors:** Zhen Guo, Chen Tian, Yang Shi, Xue-Ru Song, Wei Yin, Qing-Qing Tao, Jie Liu, Guo-Ping Peng, Zhi-Ying Wu, Yan-Jiang Wang, Zhen-Xin Zhang, Jing Zhang

**Affiliations:** 1 Department of Pathology, The First Affiliated Hospital, Zhejiang University School of Medicine, Hangzhou, 310003 Zhejiang China; 2 MOE Frontier Science Center for Brain Science and Brain-Machine Integration, School of Brain Science and Brain Medicine, Zhejiang University, Hangzhou, 310058 China; 3 Core Facilities, Zhejiang University School of Medicine, Hangzhou, 310011 China; 4 Department of Neurology and Research Center of Neurology in Second Affiliated Hospital, Key Laboratory of Medical Neurobiology of Zhejiang Province, Zhejiang University School of Medicine, Hangzhou, 310000 China; 5 Department of Neurology and Centre for Clinical Neuroscience, Daping Hospital, Third Military Medical University, Chongqing, 400042 China; 6 Department of Neurology, The First Affiliated Hospital, Zhejiang University School of Medicine, Hangzhou, 310003 China; 7 Department of Neurology and Clinical Epidemiology Unit, Peking Union Medical College Hospital, Chinese Academy of Medical Sciences, Beijing, 100730 China; 8 National Health and Disease Human Brain Tissue Resource Center, Zhejiang University, Hangzhou, 310012 China; 9 Liangzhu Laboratory, Zhejiang University, 311121 Hangzhou, China

**Keywords:** Alzheimer’s disease, Blood-based biomarker, Extracellular vesicles, Nanoflow cytometry, Brain regionally enriched, Differential diagnosis

## Abstract

Accurate differential diagnosis among various dementias is crucial for effective treatment of Alzheimer’s disease (AD). The study began with searching for novel blood-based neuronal extracellular vesicles (EVs) that are more enriched in the brain regions vulnerable to AD development and progression. With extensive proteomic profiling, GABRD and GPR162 were identified as novel brain regionally enriched plasma EVs markers. The performance of GABRD and GPR162, along with the AD molecule pTau217, was tested using the self-developed and optimized nanoflow cytometry-based technology, which not only detected the positive ratio of EVs but also concurrently presented the corresponding particle size of the EVs, in discovery (n = 310) and validation (n = 213) cohorts. Plasma GABRD^+^- or GPR162^+^-carrying pTau217-EVs were significantly reduced in AD compared with healthy control (HC). Additionally, the size distribution of GABRD^+^- and GPR162^+^-carrying pTau217-EVs were significantly different between AD and non-AD dementia (NAD). An integrative model, combining age, the number and corresponding size of the distribution of GABRD^+^- or GPR162^+^-carrying pTau217-EVs, accurately and sensitively discriminated AD from HC [discovery cohort, area under the curve (AUC) = 0.96; validation cohort, AUC = 0.93] and effectively differentiated AD from NAD (discovery cohort, AUC = 0.91; validation cohort, AUC = 0.90). This study showed that brain regionally enriched neuronal EVs carrying pTau217 in plasma may serve as a robust diagnostic and differential diagnostic tool in both clinical practice and trials for AD.

**Supplementary Information:**

The online version contains supplementary material available at 10.1186/s40478-024-01727-w.

## Introduction

Dementias, especially Alzheimer’s disease (AD), presents a significant burden to patients and their families, and the healthcare system, particularly as the aging population continues to grow worldwide [2, 55]. Currently, the diagnosis of AD can barely be made until the middle or advanced stages of the disease’s progression due to the insidious onset of AD [8]. Furthermore, the early symptoms of various dementias frequently manifest resemblances, thereby rendering the process of distinguishing them difficult. Moreover, it is not an uncommon occurrence for multiple types of dementias to coexist within a single patient [50], further complicating the diagnostic process.

Early and accurate diagnosis of AD and related dementias is crucial, but current methods such as brain amyloid β (Aβ)- and tau-PET have limitations due to their relatively high cost and limited availability [6, 39]. The clinical detection of other types of dementia pathology like α-synuclein in dementia with Lewy bodies (DLB) is unreliable to date. This not only impedes early diagnosis of AD and the differentiation between non-Alzheimer’s dementia (NAD) and AD, but also limits the differential diagnosis of multiple dementia pathologies occurring in the same brain. Analysis of cerebrospinal fluids (CSF) for AD-related proteins, including Aβ [23, 36], tau species (Tau and various pTau) [3, 29] and neurofilament light chain (NfL) [31] is more accessible than PET scans, but less acceptable to patients of lumbar puncture [49]. Recently, blood biomarkers have been investigated for AD diagnosis, including Aβ42/Aβ40, tau species and NfL [25, 35–37]. Plasma pTau217 and pTau181 have also been measured in other neurodegenerative diseases, in addition to AD [4, 30, 32, 33, 35, 44]. Nevertheless, the capacity of these markers to discriminate AD from other forms of dementia remains ambiguous, as blood biomarkers originate from various organs and systems, thereby introducing confounding effects that fail to accurately reflect alterations in the central nervous system (CNS) and impede the evaluation of neurogenic biomarkers [16, 18]. More specifically, concerning blood pTau217, a marker showing significant promise in AD screening assays, Mielke et al. observed that pTau217 could only moderately predict abnormal entorhinal cortex tau PET (AUC = 0.81) [33]. Likewise, in another study, pTau217 alone demonstrated comparable efficacy in predicting abnormal amyloid and tau PET with AUC values of 0.639–0.737 and 0.754–0.774, respectively [32]. Consequently, the advancement of economical, rapid, and convenient detection methods for plasma biomarkers undeniably holds the potential to significantly propel the clinical diagnosis and treatment practices of AD.

Mounting evidence indicates that extracellular vesicles (EVs) possess the capacity to transport distinct molecular information, which is intricately shaped by factors like the originating cell type and its physiological state. Pertinently, alterations in the composition, quantity, and size of EVs during pathological conditions can underpin the establishment of disease-specific biomarkers for precise diagnoses [47, 53]. Previous studies have established that EVs originating from the CNS can traverse the blood–brain barrier, entering the peripheral blood [15, 42], thereby facilitating bidirectional information exchange between the central and peripheral systems. Recently, the strategy of detecting CNS-specific biomarkers from the blood by targeting EVs, including exosomes, derived from the CNS, or even from neurons or glia specifically, has gained attention [15, 42]. For example, previous studies have demonstrated that the level of NMDAR2A, a protein involved in synaptic function, in plasma EVs could serve as a biomarker for synaptic dysfunction in AD [50]. Furthermore, Goetzl et al. [13] and Agliardi et al. [1] found the reduced levels of neuronal proteins with known synaptic functions in plasma neural-derived exosomes (NDEs) of AD patients compared to healthy controls (HC). Lately, Manolopoulos et al. identified plasma neuron-derived extracellular vesicles-associated Aß42/40 ratio and proBDNF (with good correlations with MMSE scores) as possible biomarkers for AD diagnosis and monitoring [28]. Kumar et al. found that miRNA of brain cell-derived small extracellular vesicles correlated with the temporal cortical region thickness on magnetic resonance imaging (MRI) and could serve as a novel blood-based molecular biomarker for AD [26]. However, differentiating AD from NAD using plasma neural-derived EVs markers remains challenging. To address this issue, a potential approach is to make use of the known differences between NAD pathologies, e.g., focusing on EVs derived from specific brain regions such as the hippocampus and cortex, which are closely involved in the development and progression of AD, especially the hippocampal regions are altered during the initial phases of the disease [16]. This may potentially pave the way for the identification of biomarkers that demonstrate a more robust correlation with the underlying pathology, thus enhancing precision diagnostics of diverse types of dementias.

Here, we systematically explored new neuronal EVs markers enriched in the hippocampus and cortex and optimized a fast and highly sensitive nanoflow cytometry-based assay with a 30 nm detection limit to assess their specificity in differentiating AD from NAD and HC. The samples utilized in the analysis were obtained from various medical centers and underwent independent confirmation of AD versus NAD diagnosis through CSF and/or PET testing.

## Methods and materials

### Participants and sample collection

Participating centers obtained ethics approval before enrollment in the study, and all participants provided written informed consent before blood collection. Samples were collected from a total of 523 participants in the multicenter study. The discovery cohort consisted of 132 patients with AD, 93 patients with NAD, and 85 HC, all meeting the clinical inclusion criteria and enrolled in the First Affiliated Hospital, Zhejiang University School of Medicine, and Peking Union Medical College Hospital. All participants underwent comprehensive clinical evaluation with the criteria described previously [40, 41]. The selected AD patients had CSF molecular signature and/or PET patterns consistent with AD based on previous cutoffs [17, 20, 50]; subjects with NAD exhibited clinical symptoms of dementia, but did not show molecular/PET evidence of AD; HC had normal clinical evaluations and majority did not have CSF/PET data (Table 1). Sample collection and plasma separation were also performed as previously described [43]. Regarding the classification of different dementias with CSF, it is important to note that the binding capacity of tau antibodies may vary depending on the nature of tau species, as well as the presence or absence of mutations, which occur in certain NAD cases. However, it is widely acknowledged that the absence of characteristic changes in CSF biomarkers effectively excludes the diagnosis of AD in each individual. In fact, a few years ago, the National Institute on Aging and Alzheimer’s Association (NIA-AA) introduced an “ATN classification system” that serves as the “gold standard” for evaluating dementia patients’ biomarker-based and biological definitions [22].

**Table 1:** Characteristics of the discovery and validation cohorts

	Discovery cohort (n = 310)			Validation cohort (n = 213)		
	HC	AD	NAD	HC	AD	NAD
Number of cases	85	132	93	70	99	44
Gender						
Female	54 (63.5%)	68 (51.5%)	34 (36.6%)	30 (42.9%)	70 (70.7%)	16 (36.4%)
Male	31 (36.5%)	64 (48.5%)	59 (63.4%)	40 (57.1%)	29 (29.3%)	28 (63.6%)
Age (years)						
Mean ± SD	64.69 ± 13.14	66.72 ± 9.064	64.20 ± 9.125	53.59 ± 15.55	63.76 ± 10.81	61.66 ± 9.183
Range	45–85	46–86	43–84	37–80	47–84	49–85
MMSE						
Mean ± SD	28.81 ± 1.569	13.33 ± 7.578	24.26 ± 6.035	28.04 ± 1.706	14.70 ± 6.805	17.95 ± 8.618
Range	22–30	0–30	3–29	25–30	0–28	0–28
	–	–	–	MoCA	–	–
Mean ± SD	–	–	–	25 ± 2.070	8.391 ± 5.698	12.23 ± 7.549
Range	–	_	–	22–28	0–25	0–26
	CSF t-tau protein (pg mL^−1^) (Lumipulse)			Duration of disease (years)		
Mean ± SD	236.8 ± 73.54	468.0 ± 240.9	213.0 ± 66.61	–	2.605 ± 2.561	2.893 ± 1.745
Range	107.0–329.0	191.0–1372	107.0–420.0	–	0.5–12	0.5–6
	CSF p-tau181 protein (pg mL^−1^) (Lumipulse)			PIB-PET (Centiloid)		
Mean ± SD	31.13 ± 11.88	75.97 ± 44.48	26.70 ± 11.30	1.607 ± 9.060	71.30 ± 22.52	− 0.4790 ± 10.26
Range	14.00–51.00	14.00–203.0	11.00–72.00	− 6.179–17.01	13.02–132.3	− 32.46–17.15
	CSF t-tau/Aβ42 (Lumipulse)			CSF t-tau protein (pg mL^−1^)		
Mean ± SD	0.9503 ± 0.5496	3.416 ± 2.528	1.079 ± 0.7243	153.0 ± 62.51	685.8 ± 747.7	319.9 ± 103.8
Range	0.3920–2.150	0.3477–14.47	0.4694–4.292	78.76–308.4	199.9–2343	215.1–422.4
	CSF p-tau181/Aβ42 (Lumipulse)			CSF Aβ40 protein (pg mL^−1^)		
Mean ± SD	0.1146 ± 0.09567	0.5698 ± 0.4433	0.1203 ± 0.07630	17,169 ± 4689	11,664 ± 8710	13,603 ± 7347
Range	0.04612–0.3643	0.05263–2.528	0.05943–0.5926	12,130–29,803	738.8–26,699	5947–20,596
	CSF Aβ40/Aβ42 (Lumipulse)			CSF Aβ42 protein (pg mL^−1^)		
Mean ± SD	0.1080 ± 0.04268	0.06773 ± 0.02852	0.1212 ± 0.02876	1757 ± 570	442.4 ± 213.7	1118 ± 585.6
Range	0.03571–0.1810	0.02548–0.1673	0.04390–0.1772	1128–3196	213.6–860.8	444.1–1506

CSF and plasma samples were collected between 7:00 and 7:30 AM under fasting state

*HC* healthy control, *AD* Alzheimer’s disease, *NAD* non-AD dementia, *SD* standard deviation, *MMSE* mini-mental state examination, *MoCA* Montreal Cognitive Assessment, *CSF* cerebrospinal fluid, *t-tau* total-tau, *Aβ40* amyloid beta 1–40, *Aβ42* amyloid beta 1–42, *PIB* Pittsburgh compound B, *PET* Positron Emission Tomography

The validation cohort consisted of 99 patients with AD, 44 patients with NAD and 70 HC, and was obtained from the Second Affiliated Hospital, Zhejiang University School of Medicine and Daping Hospital, Third Military Medical University, using the same inclusion and exclusion criteria. For assay development, reference plasma samples (n = 3, each sample was pooled from 10 healthy controls) were obtained from the First Affiliated Hospital, Zhejiang University School of Medicine as previously described [41–43].

Human brain tissue samples (3 subjects, each comprising 4 brain regions) were obtained from the National Human Brain Bank for Health and Diseases, Zhejiang University. These tissue samples were from males aged over 60 years with no apparent pathological alterations.

### Mouse cortical or hippocampal neurons culture and EVs enrichment

Primary neuron cultures were prepared from mouse (postnatal day 1) cortex or hippocampus. Briefly, after the mice were decapitated, the hippocampus and cortex were isolated, and were quickly and separately put in ice-cold dissection media (neurobasal medium containing 2% B27, 0.5 mM L-glutamine). After careful removal of all meninges, the tissues were roughly dissociated, and digested with 0.25% trypsin for 10–20 min at 37 °C. Fetal bovine serum was added to terminate the digestion reaction. After trituration, the cells were harvested by centrifugation 1000×*g* for 5 min at 4 °C and resuspended in culture media (DMEM containing 10% fetal bovine serum, 1% penicillin/streptomycin). Then, cortical and hippocampal neurons were plated onto poly-D-lysine-coated T25 flasks respectively. After 1 day, culture media was changed to neurobasal media (neurobasal medium containing 2% B27, 2 mM L-glutamine, 1% penicillin/streptomycin, and 5 µM cytosine β-D-arabinofuranoside; Cytosine β-D-arabinofuranoside exhibits the capacity to impede glial cell proliferation while concurrently augmenting neuronal population to a remarkable extent surpassing 95% [58]). The change of half media was performed on day 3, 6, 9 and 14. The B27 and fetal bovine serum used above were depleted of EVs by ultracentrifuging at 100,000×*g* for 18 h at 4 °C (Beckman Coulter Optima XPN-100 centrifuge, SW41 Ti rotor) [51]. The media (~ 50 mL), collected on day 21, was centrifuged at 2000×*g* for 20 min at 4 °C. The supernatant was ultracentrifuged at 100,000×*g* for 2 h at 4 °C (Beckman Coulter Optima XPN-100 centrifuge, SW41 Ti rotor), and the resulting pellet was resuspended with phosphate buffered saline (PBS) (pH 7.4, 0.22 μm-filtered), and ultracentrifuged again at 100,000×*g* for 2 h at 4 °C (Beckman Coulter Optima XPN-100 centrifuge, SW41 Ti rotor). The obtained pellets (fractions enriched with EVs) were resuspended with 100 μL PBS and stored at − 80 °C before use.

### Mass spectrometry

Mass spectrometry-based detection was employed to explore and identify biomarkers associated with region-specific EVs. Cortical/hippocampal derived EVs were lysed in buffer containing 8 M urea (pH 8.5) and protease inhibitor. Samples were sonicated before centrifugation at 12,000×*g* for 10 min at 4 °C. Protein concentrations of the supernatant were determined using a BCA kit. The protein (100 μg) was subsequently reduced with 5 mM DTT for 30 min at 65 °C and alkylated for 15 min with 11 mM iodoacetamide. Then the solution was replaced with 0.1 M NH_4_HCO_3_ using a 10 kD ultrafilter. Trypsin was added at a 1:50 mass ratio of trypsin to total protein and incubated overnight at 37 °C (> 12 h). Following digestion, peptides were acidified with TFA and dried with a vacuum concentrator evaporator. Then, peptides were dissolved with 0.1% TFA, desalted over a C18-stage tip, and dried again. Samples were then resuspended in 15 μL of bRP (basic reverse phase) buffer A (10 mM NH_4_HCO_3_, pH 10, 5% ACN); and 1ug peptides were analyzed on timsTOF Pro 2 (Bruker, Germany).

The MS data were processed using Proteome Discoverer software v2.5 (Thermo Fisher), and were searched against SwissProt Mouse database (17,201 sequences) using the SEQUEST algorithm. Trypsin(full) was specified as cleavage enzyme allowing up to 2 missing cleavages. The minimum peptide length was 6 amino acids with a maximum of 5 modifications per peptide. The mass tolerance for precursor ions was set as 10 ppm, and the mass tolerance for fragment ions was set as 0.02 Da. Carbamidomethyl on Cys was specified as fixed modification. The oxidation of Met (M), the acetyl, met-loss, and met-loss + acetyl of protein N-terminal were set as dynamic modifications. Proteins and peptide-spectrum matches (PSMs) were filtered with a maximum false discovery rate (FDR) of 1%.

### EV enrichment from plasma sample

EVs were enriched from plasma utilizing an ultracentrifugation methodology. Frozen plasma samples were thawed quickly at 37 °C and centrifuged at 2000×*g* for 15 min at 4 °C to obtain platelet-free plasma, followed by 12,000×*g* for another 30 min at 4 °C to remove large cell debris. The supernatant (100 μL) was further diluted with PBS (pH 7.4, 0.22 μm-filtered) at a ratio of 1:10 and then ultracentrifuged at 100,000×*g* for 1 h at 4 °C (Beckman Coulter Optima MAX-XP centrifuge, TLA-55 rotor). The pellet was resuspended with PBS, and ultracentrifuged again at 100,000×*g* for 1 h at 4 °C. The obtained pellets (EV-enriched fractions) were resuspended with 100 μL PBS and stored at − 80 °C before use. EVs-depleted plasma supernatant was used to test the specificity of the NanoFCM assay.

### Immunohistochemistry

IHC examination was performed on 4 μm paraffin-embedded sections (cortex, hippocampus, caudate, and cerebellum) and stained with a Ventana BenchMark staining device (Ventana Medical Systems Inc). Slides were dried at 60 °C for 1 h and then deparaffinized with EZ Prep (Ventana, 950-102) for 15 min. Endogenous peroxidases were blocked for 10 min with a 3.0% hydrogen peroxide solution from the OptiView DAB IHC Detection Kit (Ventana, 760-700). Then, slides were heated to 100 °C for 36 min in ULTRA Cell Conditioning Solution 1 (Ventana, 950-224). Primary anti-GABRD rabbit polyclonal antibody (Invitrogen, PA5-26307, 1:100 dilution); anti-GPR162 rabbit polyclonal antibody (Proteintech, 15254-1-AP, 1:200 dilution), anti-NeuN mouse monoclonal antibody (Sigma-Aldrich, MAB377, 1:200 dilution) and anti-Histone H3 rabbit polyclonal antibody (Abcam, ab5103, 1:300 dilution) were diluted in Tris buffered antibody diluent (pH 7.2, 15 mM NaN3 and stabilizing protein, Dako, Santa Clara, CA, USA) followed by overnight incubation at 4 °C. Visualization was performed using the OptiView DAB IHC Detection Kit (Ventana, 760-700) followed by nuclear counterstaining by hematoxylin II (Ventana, 790-2208), bluing staining (Ventana, 760-2037), dehydration, transparency, and mounting.

### Electron cryo-microscopy

EV-enriched fractions from reference plasma (EVs fractions; 5 μL) were deposited on electron microscopy (EM) grids coated with perforated carbon film for 5 min; and plunge-frozen in liquid ethane using a Vitrobot (Thermo Fisher). Images were acquired on a Talos F200C (Thermo Fisher) operating at 200 kV.

### Nanoparticle Tracking Analysis

We utilized the conventional Nanoparticle Tracking Analysis (NTA) method to characterize the distribution and quantify the abundance of EVs in plasma. NTA was performed using a NanoSight NS300 with a 405 nm violet laser (Malvern, UK) according to the manufacturer’s instructions. Briefly, ultracentrifuged reference plasma EVs were diluted with PBS (pH 7.4, 0.22 μm-filtered) to 1 × 10^8^–1 × 10^9^ EVs mL^−1^ with a final volume of 1 mL for direct scattering measurement. For each measurement, videos of 3 random views were captured with the following settings: temperature controller, on; temperature, 25 °C; camera level, 14; Syringe speed, 40 μL s^−1^; capture duration, 60 s. The videos were analyzed using NanoSight NTA 3.4 at automatic mode with the detection threshold of 5 to assess mean and modal particle diameters, D50 values and particle number concentration.

### Western blot

EVs were lysed in RIPA buffer. After centrifugation at 14,000×*g* for 10 min, the supernatants were collected, mixed with SDS sample buffer, and boiled for 5 min. The protein samples were subjected to SDS–Polyacrylamide Gel Electrophoresis (SDS-PAGE) and transferred to polyvinylidene difluoride membranes. The criterion employed for loading the gels with equivalent quantities relied upon the overall protein concentration. After blocking with 5% nonfat milk, the membranes were incubated with primary antibodies overnight at 4 °C, followed by incubation with IRDye 800CW secondary antibody (LI-COR) [diluted in Tris Buffered Saline with Tween 20 (TBST)]. The blots were visualized by Odyssey CLx Imaging System (LI-COR). The primary antibodies used in the present study included anti-GABRD antibody (Proteintech, 15623-1-AP), anti-GPR162 antibody (Proteintech, 15254-1-AP), anti-pTau217 antibody (Invitrogen, 44-744), anti-TSG101 antibody (Proteintech, 67381-1-Ig), anti-CD9 antibody (Proteintech, 60232-1-Ig) and anti-albumin antibody (Proteintech, 16475-1-AP). Primary antibodies were diluted in universal antibody diluent (NCM biotech, WB100D).

### Stochastic optical reconstruction microscopy

Zenon immunoglobulin G (IgG) labeling kits (Invitrogen) were used to prepare fluorophore-conjugated antibodies according to the manufacturer’s instructions. For stochastic optical reconstruction microscopy (STORM) experiments, anti-GABRD antibody (Proteintech, 15623-1-AP) or anti-GPR162 antibody (Proteintech, 15254-1-AP) was labeled with Zenon™ Alexa Fluor™ 488 rabbit IgG Labeling Kit (Invitrogen, Z25302), anti-pTau217 antibody (Invitrogen, 44-744) was labeled with a Zenon™ Alexa Fluor™ 647 rabbit IgG Labeling Kit (Invitrogen, Z25308). Ultracentrifuged plasma (EVs fractions; 10 μL) was thawed and blocked with an equal volume of 2% BSA for 1 h at room temperature before diluted with 10 μL PBS (pH 7.4, 0.22 μm-filtered). Labeled anti-GABRD antibody or anti-GPR162 antibody (0.06 μg), anti-pTau217 antibody (0.1 μg), together with PE anti-human CD9 antibody (0.06 μg) (BioLegend, 312106) were added to the blocked EVs sample and incubated overnight at 4 °C. The labeled sample was then fixed with 20 μL 4% PFA (0.22 μm-filtered) for 20 min at room temperature. Labeled EVs were washed three times with PBS (pH 7.4, 0.22 μm-filtered) and 200 μL of specialized STORM imaging buffer [7 μL of oxygen-scavenging GLOX buffer (14 mg of glucose oxidase, 50 μL of 17 mg mL^−1^ catalase in 200 μL of 10 mM Tris, 50 mM NaCl, pH 8.0), 70 μL of MEA buffer (1 M), plus 620 μL of Buffer B (50 mM Tris–HCl (pH 8.0), 10 mM NaCl, 10% Glucose)] was added before image acquisition with a STORM. All images were acquired on a Nikon N-STORM super-resolution system (Nikon Instruments Inc.) with a Nikon Eclipse Ti inverted microscope with a 100 × TIRF lens (numerical aperture 1.49). 2000 frames with a 60 ms exposure time were recorded to image one field by an electron multiplying CCD camera (Andorixon DU-897). During the fluorescence acquisition, Nikon microscopic imaging device provided a Perfect Focus System (PFS) to achieve real-time correction of focus drift in Z-axis direction.

### EV analysis with NanoFCM Flow NanoAnalyzer

A NanoFCM Flow NanoAnalyzer (NanoFCM Inc., XiaMen, China), which readily detects 30–1000 nm nanoparticles, was used to analyze particle concentration, size distribution and protein marker phenotyping according to the manufacturer's instructions and reported protocols [52]. Two single photon counting avalanche photodiodes (APDs) were used for the simultaneous detection of side scatter (SSC) (FF01-488/6 bandpass filter for a 488 nm laser or a FF01-524/24 bandpass filter for a 532 nm laser) and fluorescence (FF01-525/45 bandpass filter for green fluorescence, FF01-579/34 bandpass filter for orange fluorescence, or FF01-630/69 bandpass filter for red fluorescence) of individual particles. The 250 nm PE and AF488 fluorophore-conjugated polystyrene beads of known concentration were used to calibrate the sample particle concentration. The Silica Nanosphere Cocktail (NanoFCM Inc., S16M-Exo) that contained a mixture of 68 nm, 91 nm, 113 nm and 155 nm beads were used as the particle size standards to test the size distribution of EVs. Particles passed by the detector during a 1 min interval were recorded in each test. Samples were diluted to attain a particle count within the optimal range of 4000–8000 min^−1^. Using the calibration curve, the flow rate and side scattering intensity were converted into corresponding vesicle concentration and size on the NanoFCM software (NanoFCM Profession V1.0).

Fluorophore-conjugated antibodies were generated as mentioned above. In short, anti-GABRD antibody (Proteintech, 15623-1-AP), or anti-GPR162 antibody (Proteintech, 15254-1-AP), or anti-NLGN3 antibody (Abcam, ab192880) was labeled with Zenon™ Alexa Fluor™ 488 rabbit IgG Labeling Kit (Invitrogen, Z25302); anti-pTau217 antibody (Invitrogen, 44-744) was labeled with a Zenon™ Alexa Fluor™ 647 rabbit IgG Labeling Kit (Z25308, Invitrogen).

For optimization of Nanoscale flowcytometry assays experiment, immunoglobulin isotype controls of corresponding species were also labeled at the same final concentrations as all the antibodies. Another negative control (no antibody “Blank”, i.e., dye only) was done with the same volume of PBS instead of specific antibodies during the labeling reaction. EVs, or EVs-depleted plasma, or PBS (10 μL) were blocked with an equal volume of 2% BSA for 1 h at room temperature before diluted with 10 μL PBS (pH 7.4, 0.22 μm-filtered). Then, the blocked EVs, or EVs-depleted plasma, or PBS were incubated with fluorophore-conjugated antibodies [anti-GABRD antibody (0.06 μg), or anti-GPR162 antibody (0.06 μg), or anti-NLGN3 antibody (0.06 μg), or anti-pTau217 antibody (0.1 μg)], or corresponding IgG isotype control, or dye, overnight at 4 °C. The labeled sample was then fixed with 20 μL 4% PFA (0.22 μm filtered) for 20 min at room temperature, followed by analyzing with NanoFCM. The samples were diluted linearly (final volumes of 50 μL, 100 μL and 150 μL, respectively) to evaluate the accuracy of the assays. And the day-to-day stability was evaluated with a single reference EV sample that was analyzed in duplicate and repeated over five days.

For cohort study, the blocked EVs were incubated with fluorophore-conjugated antibodies [anti-GABRD antibody (0.06 μg), or anti-GPR162 antibody (0.06 μg), together with anti-pTau217 antibody (0.1 μg)] overnight at 4 °C. The labeled sample was then fixed with 20 μL 4% PFA (0.22 μm-filtered) for 20 min at room temperature, followed by analyzing with NanoFCM. All samples were kept at 4 °C and tested within 8 h after labeling, and labeling was stable under these conditions. A reference plasma sample was added into each day’s measurements to help to assess day-to-day variations (≤ 10%).

### Ethics oversight

The study was performed in accordance with the ethical standards as laid down in the 1964 Declaration of Helsinki and its later amendments for experiments involving humans. Meanwhile, all human sample studies, including those for the Cryo-EM, NTA, WB, STORM and NanoFCM study protocols, were approved by the clinical research ethics committees of the First Affiliated Hospital, College of Medicine, Zhejiang University (approval number: 2021-400). The IHC protocol for human brain investigations was approved by the clinical research ethics committees of the First Affiliated Hospital, College of Medicine, Zhejiang University (approval number: 2022-043). The experiment involving mice was approved by the animal experimental ethical inspection committees of the First Affiliated Hospital, College of Medicine, Zhejiang University (approval number: 2021-667) and performed in accordance with Chinese Laboratory Animal Guideline for ethical review of animal welfare (2018/09) for the care of laboratory animals.

### Statistical analysis

All analyses were performed with SPSS 25.0 (IBM, Chicago, IL, USA) or Prism 8.0 (GraphPad Software, La Jolla, CA, USA). The Mann–Whitney U test (for two groups) or the one-tailed nonparametric ANOVA, Kruskal–Wallis test (for three groups) were used to compare the mean total number of particles detected by scatter, or ratio of a given positive marker to total events. Receiver Operating Characteristic (ROC) curves were generated to evaluate their sensitivity and specificity in distinguishing AD from HC or NAD. Logistic regression was used to create an integrative model that included multiple plasma biomarkers. The bootstrap method was used to estimate a 95% confidence interval through 1000 sampling iterations. Delong’s test was used to confirm whether the integrated model has a significantly different AUC from single-factor diagnostic model. *p* < 0.05 was regarded as significant.

## Results

### Characteristics of clinical cohorts

The clinical cohort included in this study was sourced from multiple centers and collected based on standardized criteria. Furthermore, a substantial amount of clinical and radiological imaging/CSF information was collected in conjunction with the samples. The multi-center study enrolled a total of 523 participants, consisting of a discovery cohort and a validation cohort (Table 1). Based on the levels of Aβ40, Aβ42, t-tau, p-tau in CSF and/or Centiloid scale of PIB-PET, participants with cognitive impairment included were classified as AD or NAD. For several logistic reasons, most HC subjects did not have CSF/PET data (see the Method section for more details); thus, clinically cognitive normal subjects were grouped as HC. The discovery cohort consisted of 132 patients with AD (mean age ± SD, 66.72 ± 9.064; 68 female and 64 male), 93 patients with NAD (mean age ± SD, 64.20 ± 9.125; 34 female and 59 male), and 85 HC (mean age ± SD, 64.69 ± 13.14; 54 female and 31 male). The validation cohort consisted of 99 patients with AD (mean age ± SD, 63.76 ± 10.81; 70 female and 29 male), 44 patients with NAD (mean age ± SD, 61.66 ± 9.183; 16 female and 28 male) and 70 HC (mean age ± SD, 53.59 ± 15.55; 30 female and 40 male) (Table 1).

### Selection of the candidate markers

Clinical diagnosis of AD early and accurately is quite challenging. We previously found that NMDAR2A, together with L1CAM, can differentiate AD readily from HC [50], but the assay was much less effective in differentiating AD from NAD (Additional file 2: Fig. S1). To identify potential markers that can help with differentiation of AD from NAD, we employed extensive unbiased mass spectrometry to analyze EVs derived from mouse primary cortical and hippocampal neurons. These brain regions play a critical role in the development and progression of AD. 1574 and 825 proteins were detected by mass spectrometry from the cortical and hippocampal primary neuron derived EVs, respectively (Additional file 1: Table S1 and Table S2). Most established EVs proteins, as listed on the website http://www.microvesicles.org/, were successfully identified, thereby affirming the credibility of the methodology utilized in the approach. Among the identified proteins, three proteins related to neurodegeneration, gamma-aminobutyric acid receptor delta subunit (GABRD), G protein-coupled receptor 162 (GPR162) and Neuroligin 3 (NLGN3), were selected for further investigations (See Additional file 2: Fig. S2 for the workflow of this study). As indicated previously, to enrich neuronal EVs isolated from primary cultures, cytosine β-D-arabinofuranoside was used, which not only restrained glial cell proliferation but also facilitated the enrichment of neurons with a purity exceeding 95% [58].

To test whether these CNS EV-tagged proteins are present in plasma at appropriate levels, we examined them in EVs isolated from pooled human plasma samples with a flow cytometry-based technology developed previously by us [50], with minor adjustments. Compared to GABRD and GPR162, the labeling of NLGN3 in the plasma EVs exhibited less stability, with much bigger variations (Additional file 2: Fig. S3). Additionally, we identified pTau217, a plasma marker associated with AD, in these plasma EVs through NanoFCM measurement (Fig. 3), even though its prior association with EVs had not been established.

**Fig. 1:**
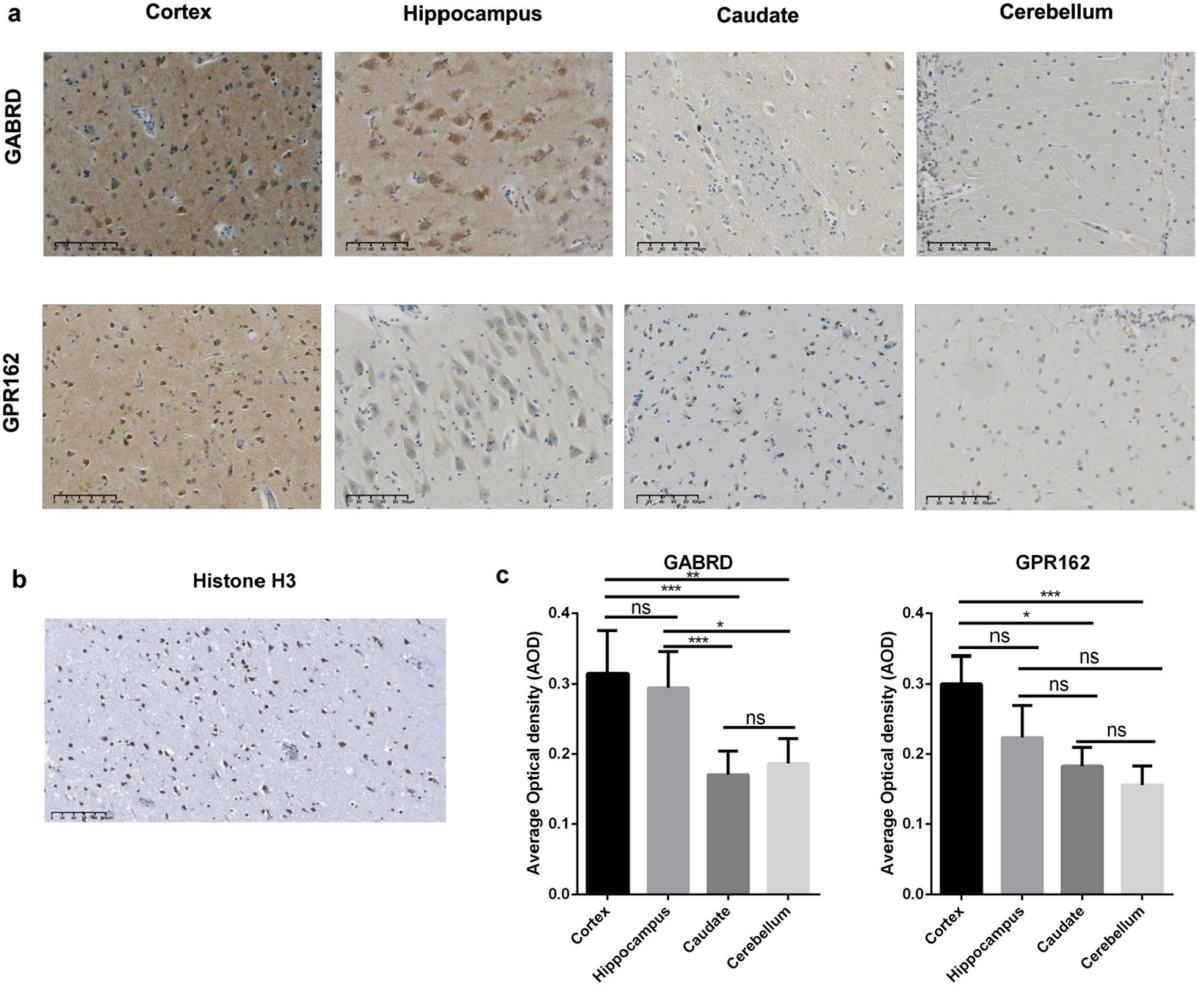
GABRD and GPR162 expression in human brain **a** IHC analysis of the cortex, hippocampus, caudate and cerebellum. GABRD and GPR162 stained positively and strongly at a subset of neurons in the cortex, hippocampus, and less robustly in the caudate and cerebellum (n = 3). Scale bar = 100 μm. **b** Histone H3 was stained as internal control, and stained positively at nucleus, rather than other cellular components. Scale bar = 100 μm. **c** Histogram showed the expression abundance of markers in each brain region. *p < 0.05, **p < 0.01, ***p < 0.001, one-tailed nonparametric ANOVA followed by Kruskal–Wallis test

According to the database (proteinatlas.org), GABRD is predominantly expressed in the hippocampus, cerebellum and caudate, while GPR162 is primarily distributed in the cortex, cerebellum and caudate. To validate their expressions in the human brain, we performed IHC analysis on brain regions of three individuals over 60 years without apparent diseases based on pathological evaluation. As shown in Fig. 1, GABRD and GPR162 were predominantly localized on the membrane and cytoplasm of neurons and were positive in neurons in the cortex, hippocampus, caudate nucleus, and cerebellum, although the staining in the caudate nucleus and cerebellum was less abundant (Fig. 1c). Notably, the expression levels of GABRD and GPR162 were not significantly different between the cortex and hippocampus (Fig. 1c), contradicting the information provided in the database. As an internal control, histone H3 exhibited positive staining specifically within the nucleus rather than other cellular components (Fig. 1b). To further corroborate the identity of the positive cells are neurons, we conducted co-staining of GABRD or GPR162 with the neuronal marker NeuN. Our findings demonstrated that both GABRD (mean ± SD, 93.60% ± 1.826%) and GPR162 (mean ± SD, 91.08% ± 1.660%) exhibited a preferential expression in neurons (Additional file 2: Fig. S4). Specifically, 69.35% ± 7.151% and 67.28% ± 8.204% of neurons expressed GABRD and GPR162, respectively (Additional file 2: Fig. S4). In contrast, their expression levels in non-neuronal cell types such as astrocytes, oligodendrocytes, or microglia (all NeuN negative) were significantly lower (Additional file 2: Fig. S4).

### Characterization of EVs labeled by GABRD and GPR162

**Fig. 2:**
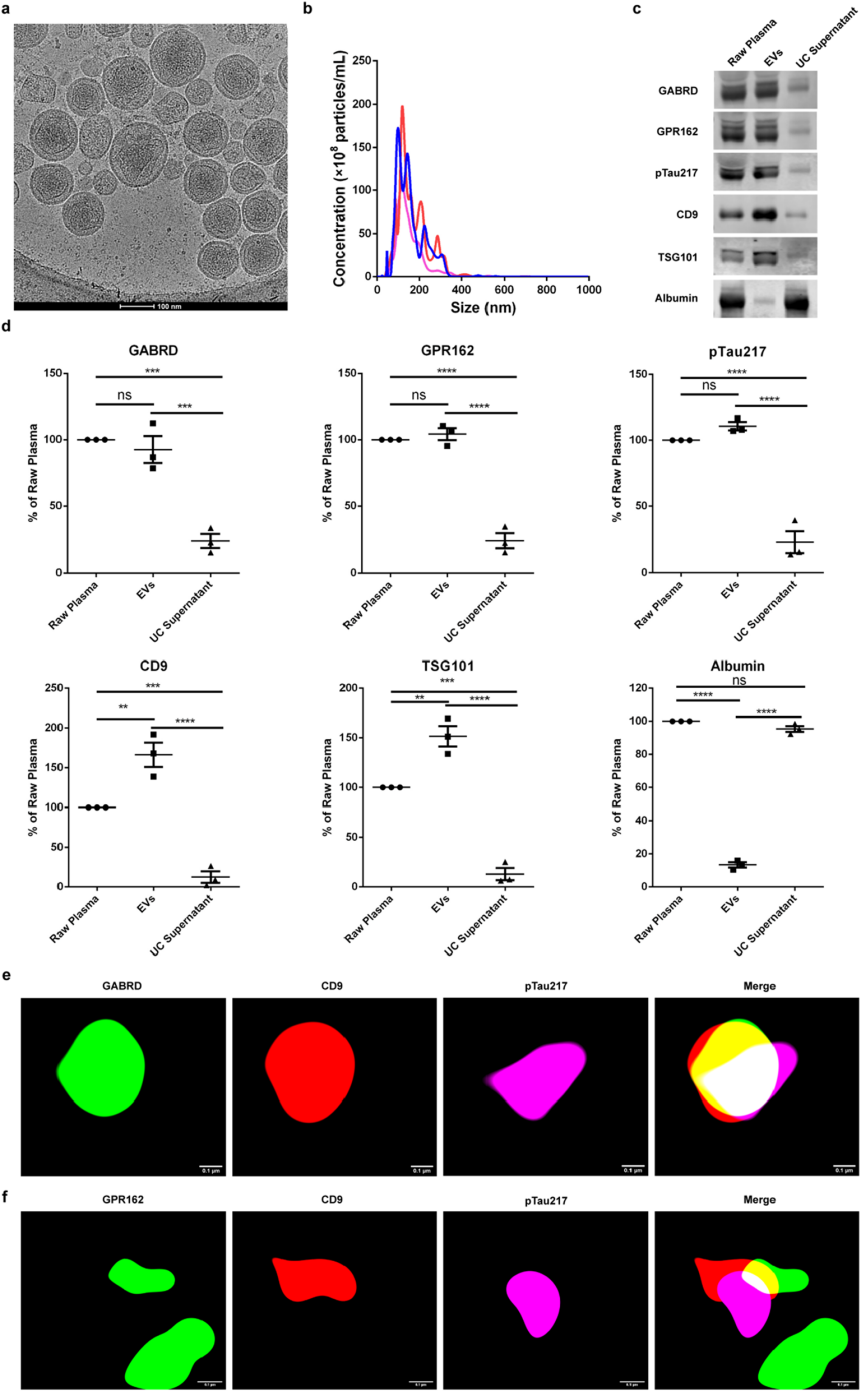
Characterization of EVs enriched by ultracentrifugation **a** EVs structure revealed by cryo-EM showed double layered membrane-bound vesicles with a diameter ≈100 nm. **b** NTA showed a population of EVs with a peak ≈100 nm (n = 3). Three lines with different colors represent three replicate experiments. **c** EVs and neuron marker proteins were present in the EVs fraction obtained by ultracentrifugation; meanwhile, the non-EVs proteins, albumin was absented in the EVs fraction. **d** Graph summarized the relative content of EVs/neuron marker proteins and non-EVs proteins of WB experiments (n = 3). **e** STORM imaging of neuron marker GABRD (green) with EVs marker CD9 (red) and pTau217 (violet) presence of EVs membranes together. Scale bar = 0.1 μm. **f** STORM imaging was performed to confirm GPR162 (green), CD9 (red) and pTau217 (violet) presence of EVs membranes together. Overlap of both markers with CD9 indicates their presence on EVs membranes. Scale bar = 0.1, μm. **p < 0.01, ***p < 0.001, ****p < 0.0001, one-tailed nonparametric ANOVA followed by Kruskal–Wallis test

Having validated the relative regional and neuronal specificity of the two markers, following the MISEV2018 guidelines [48], we characterized the plasma EVs, which were morphologically intact with lipid bilayers by cryo-EM (Fig. 2a) and had similar contents, indicating the EVs were well-preserved during enrichment procedures [50]. The diameter of the major population of EVs in the representative cryo-EM image was around 100 nm, in agreement with NTA analysis (n = 3) (Fig. 2b). The concentration of plasma EVs was 1.70 ± 0.125 × 10^11^ particles mL^−1^. Western blot was used to test the newly discovered biomarkers presenting in EVs and the results showed that the majority of GPR162, GABRD, and pTau217 were present in the EV-enriched fractions, not in the supernatant. EVs (TSG101 and CD9) and non-EVs (albumin) proteins were also tested in Western blot for quality control (Fig. 2c; the full length of the gel provided in Fig. S5). It is imperative to stress that our recent investigation has unveiled an important finding: during our EVs enrichment procedure, the bulk of high-density lipoprotein (HDL) and low-density lipoprotein (LDL) predominantly resided in the supernatant subsequent to centrifugation, rather than being concentrated within the EVs pellets [50]. Consequently, our EVs preparations were relatively free of lipoproteins.

As the average diameter of these EVs is approximately 100 nm, which is below the resolution limit of conventional confocal microscopy, STORM, a technique with 20 nm lateral resolution and 50 nm axial resolution was used to locate GABRD, GPR162 and pTau217 on EVs. STORM data revealed that GABRD and the general EV marker CD9, as well as the AD-related marker pTau217, co-localized on the surface of the same plasma EV (Fig. 2d). Similarly, colocalization of GPR162 with pTau217 and CD9, was also observed (Fig. 2e; see also Additional file 3: Video S1 and Additional file 4: Video S2 for a three-dimensional view of co-localization of GABRD/GPR162, CD9 and pTau217). Thus, by employing traditional NTA and Western blot as well as more advanced cryo-EM techniques, we have successfully confirmed the presence of abundant EVs in plasma samples enriched with ultracentrifugation, enabling us to conduct thorough detection in human plasma.

### Optimization of Nanoscale flowcytometry assays

**Fig. 3:**
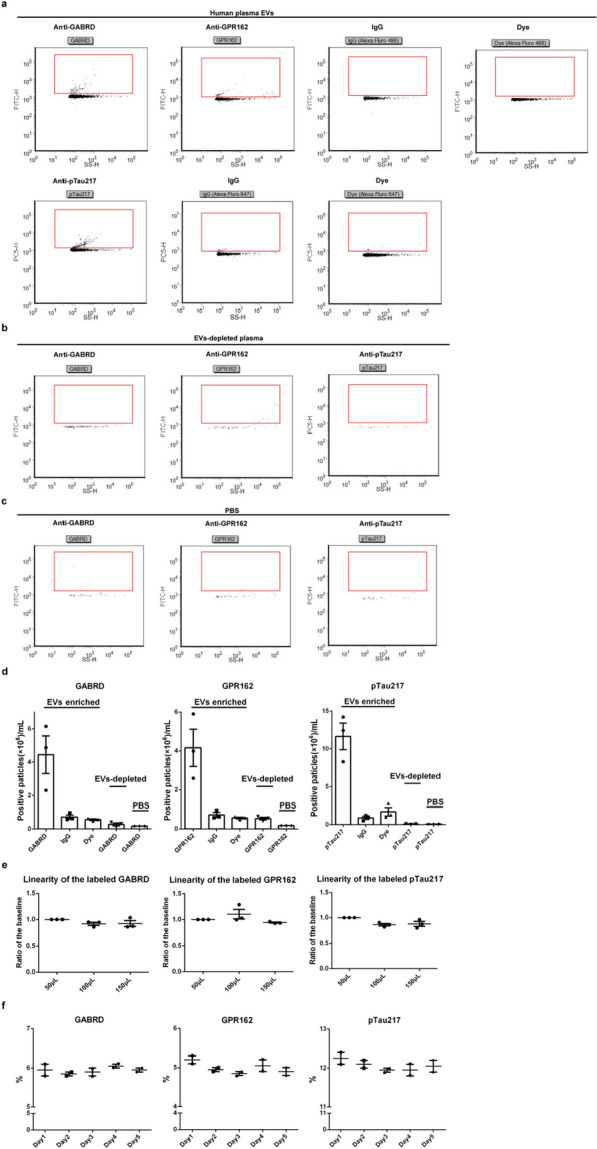
Development of novel, flow cytometry-based assays for CNS-specific and AD-associated markers on plasma EVs **a** example histograms showing populations of EVs which were positive for each marker after labeling with fluorophore-conjugated antibody; plasma EVs labeled using fluorophore-conjugated immunoglobulin G isotype control for the indicated marker target antibody; and plasma EVs incubated with dye (fluorophore only, no antibody) control experiment. **b** Histogram of remaining particles after depletion of EVs from plasma by ultracentrifugation. **c** Histogram of PBS incubated with fluorophore-conjugated antibody. **d**–**f** Summary data from experiments demonstrating specificity of EVs assays (n = 3) (**d**), linearity in different dilutions of EVs plasma samples (n = 3) (**e**), and stability of reference plasma (two replicates run each day on 5 separate days of the experiment) for GABRD, GPR162 and pTau217 (**f**). Positive particles were circled out using red boxes

Having confirmed the associations of GABRD, GPR162, and pTau217 with EVs (n = 3) (Fig. 3a–d), we focused on optimizing the flow-based assays. The day-to-day stability was evaluated with a single reference EV sample that was analyzed in duplicate and repeated over five days (Fig. 3f). The optimized assays showed relatively high accuracy (linearity of dilution) (n = 3) (Fig. 3e) and reliable reproducibility, with average within-day coefficients of variation (CV) and average day-to-day CVs ≤ 10% for all markers (Fig. 3f). Therefore, it appears that the Nanoscale flowcytometry (NanoFCM) detection method employed in this study sufficiently circumvents matrix effects and yields precise detection results.

### Sensitivity and specificity of GABRD, GPR162 and pTau217 in AD diagnosis and differential diagnosis

**Fig. 4:**
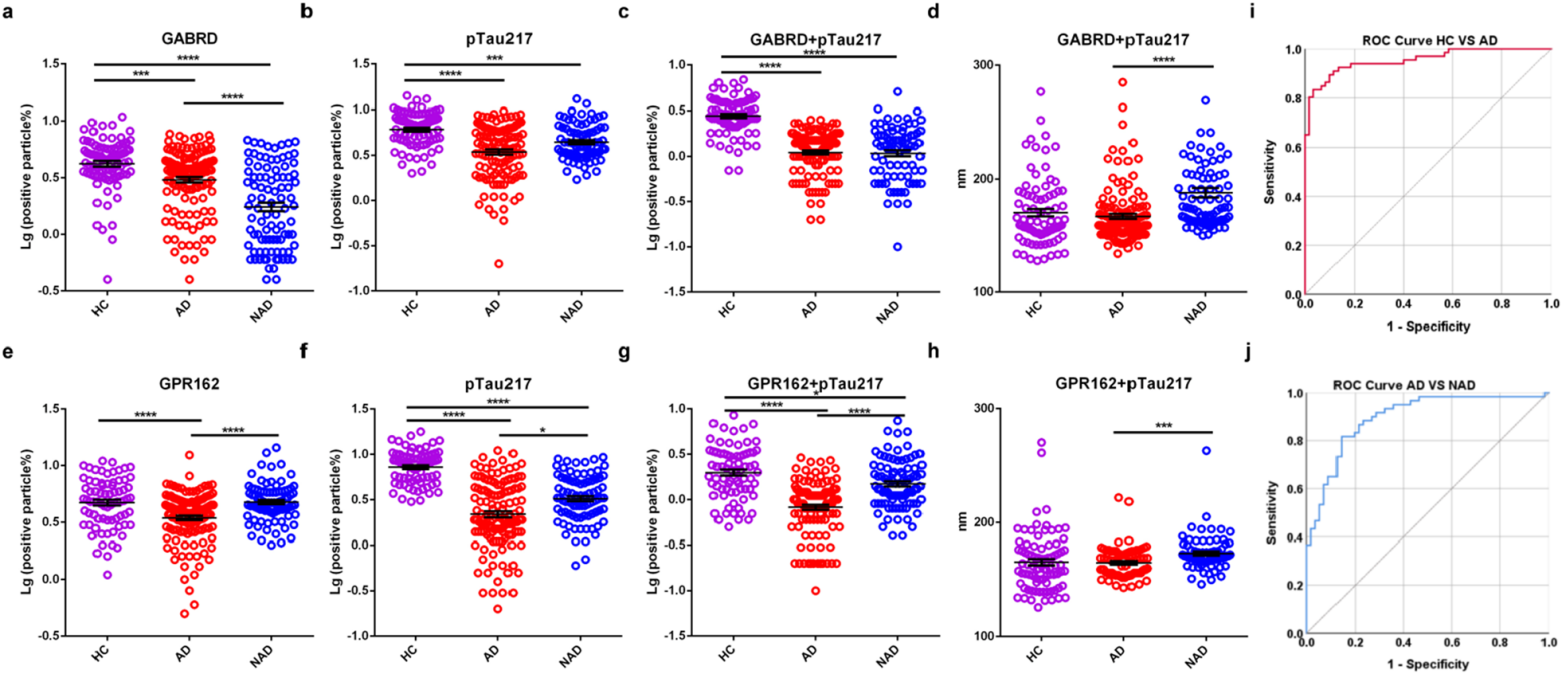
Performance of CNS-derived EVs markers in the discovery cohort **a**, **e** the ratio of GABRD^+^ or GPR162^+^ EVs in each group. **b**, **f** The ratio of pTau217^+^ EVs in each group. **c**, **g** The ratio of GABRD^+^ or GPR162^+^- carrying pTau217 EVs in each group. **d**, **h** The corresponding size of distribution mode of GABRD^+^ or GPR162^+^-carrying pTau217 EVs in each group. **i** Integrative model combining age, the ratio of GABRD^+^ or GPR162^+^-carrying pTau217 EVs, the corresponding size of distribution mode of GABRD^+^ or GPR162^+^-carrying pTau217 EVs, distinguished AD from HC. **j** Integrative model distinguished AD from NAD. *p < 0.05, ***p < 0.001, ****p < 0.0001, one-tailed nonparametric ANOVA followed by Kruskal–Wallis test

To explore nanoflow cytometry techniques in differentiating AD from HC or NAD, samples from multiple medical centers were analyzed. We examined individual samples in the discovery cohort meeting the previous CSF cutoffs [20, 40] or according to PET results to define AD or NAD. We began with a logarithmic transformation (Lg) of the labeling ratio of all markers to satisfy a normal distribution and found that the numbers of GABRD^+^, GPR162^+^ and pTau217^+^ EVs were fewer in AD compared to HC in the discovery cohort (Additional file 2: Table S3; Fig. 4a, b, e, f). When comparing AD with NAD subjects, the numbers of GPR162^+^, pTau217^+^ EVs were fewer in AD than those in NAD, while the number of GABRD^+^ EVs was the opposite (Fig. 4a–b, e–f). We also assessed EVs positive for both pTau217 and either GABRD or GPR162, finding that both GABRD^+^ and GPR162^+^-carrying pTau217 EVs were significantly fewer in AD compared to HC. Of note, significantly more GPR162^+^-carrying pTau217 EVs were detected in NAD than AD, although no significant difference was found in GABRD^+^-carrying pTau217 EVs (Fig. 4c, g).

For diagnostic sensitivity and specificity, we evaluated the performance of all markers in discriminating AD from HC or NAD using ROC analysis, and the AUC, along with the 95% confidence interval (CI) and the cut off, are presented in Additional file 2: Table S4. In comparing AD with HC, the sensitivity and specificity were 91.53% and 81.25% (AUC = 0.9166, 95% CI 0.8740–0.9592) for GABRD^+^-carrying pTau217 EVs and 78.23% and 76% (AUC = 0.8183, 95% CI 0.7546–0.8820) for GPR162^+^-carrying pTau217 EVs (Additional file 2: Table S4). For AD vs NAD, the performance was moderate (Additional file 2: Table S4).

This nanoflow cytometer (NanoFCM) not only detected the positive ratio of EVs but also concurrently presented the corresponding particle size of the EVs. Notably, besides the differences in total numbers of labeled EVs, we observed a significant difference in the size distribution of GABRD^+^/GPR162^+^-carrying pTau217 EVs, between AD and NAD patients. To further distinguish between AD and NAD, we introduced the concept of EVs size distribution, using the “mode” (i.e., the size corresponding to the most distributed number of particles) to represent the variation of particle sizes in different groups. The corresponding size of the distribution mode of GABRD^+^ or GPR162^+^-carrying pTau217 EVs was significantly smaller in AD vs NAD (Fig. 4d, h, See Additional file 2: Fig. S6 for the pattern of particle size variation). An integrative model, combining age, the corresponding size of the distribution mode of GABRD^+^ or GPR162^+^-carrying pTau217 EVs, and the ratio of GABRD^+^ or GPR162^+^-carrying pTau217 EVs, discriminated AD from NAD with an AUC of 0.91 (95% CI 0.839–0.954; sensitivity = 81.67%, specificity = 85.71%) (Fig. 4j). Furthermore, the integrated model performed even better in differentiating AD from HC, with an AUC of 0.96 (95% CI 0.921–0.988; sensitivity = 83.33%, specificity = 95% (Fig. 4i).

The aforementioned outcomes suggested that employing a single factor has indeed demonstrated relatively limited discriminatory efficacy when distinguishing between HC and AD, as well as between AD and NAD. However, the integrated model we established based on multiple factors, facilitated precise and efficient differentiation between AD and HC, as well as NAD.

**Fig. 5:**
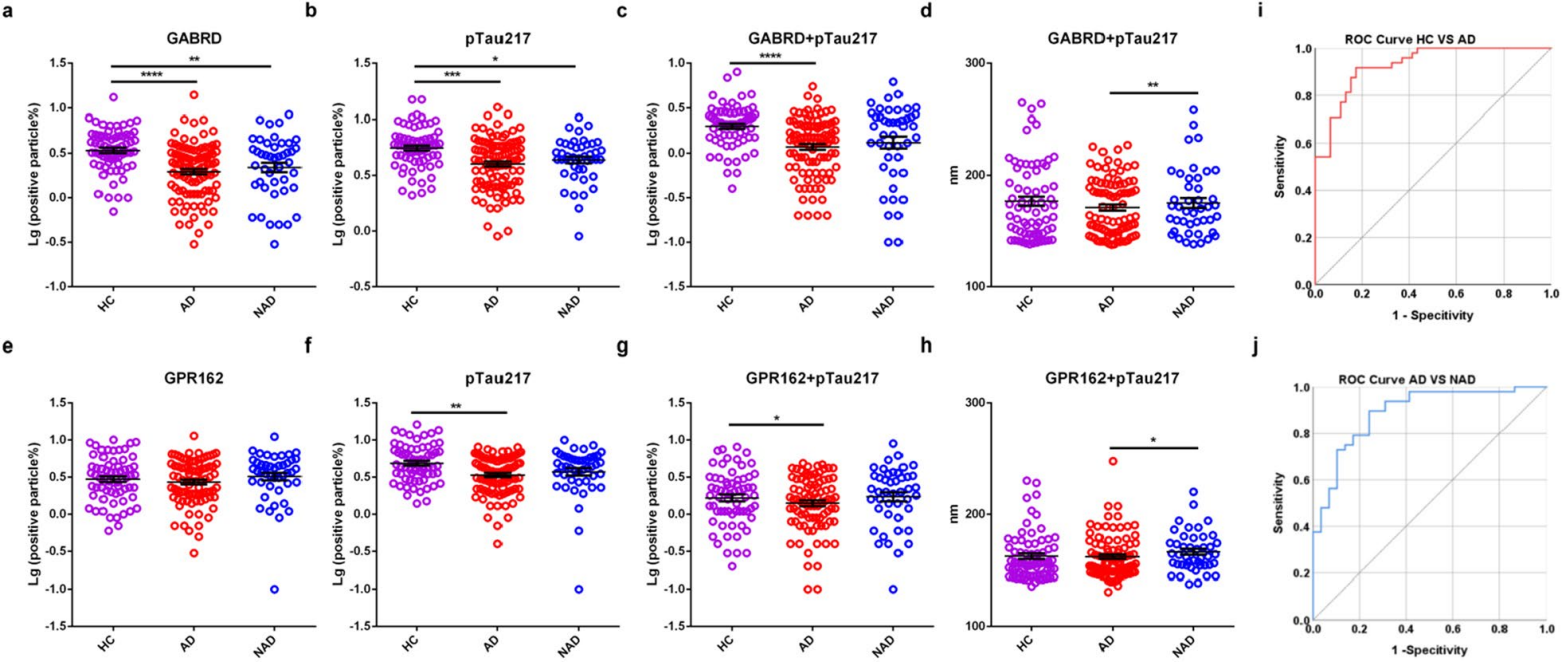
Performance of CNS-derived EVs markers in the validation cohort **a**, **e** the ratio of GABRD^+^ or GPR162^+^ EVs in each group. **b**, **f** The ratio of pTau217^+^ EVs in each group. **c**, **g** The ratio of GABRD^+^ or GPR162^+^-carrying pTau217 EVs in each group. **d**, **h** The corresponding size of distribution mode of GABRD^+^ or GPR162^+^-carrying pTau217 EVs in each group. **i** Integrative model distinguished AD from HC. **j** Integrative model distinguished AD from NAD. *p < 0.05, **p < 0.01, ***p < 0.001, ****p < 0.0001, one-tailed nonparametric ANOVA followed by Kruskal–Wallis test

To validate the diagnostic results obtained from the discovery cohort, we employed an independent validation cohort. The numbers of GABRD^+^ EVs and pTau217^+^ EVs were significantly fewer in AD vs. HC, and the number of GPR162^+^ EVs was also fewer in AD vs. HC, although a significant difference was not reached (Additional file 2: Table S3; Fig. 5a, b, e, f). Compared to NAD cases, numbers of GABRD^+^, GPR162^+^, and pTau217^+^ EVs were fewer in AD, but again did not reach a statistically different level (Fig. 5a, b, e, f). Furthermore, when evaluating the numbers of GABRD^+^ or GPR162^+^-carrying pTau217 EVs, we found that the numbers of either GABRD^+^-carrying pTau217 EVs or GPR162^+^-carrying pTau217 EVs were significantly fewer in AD than HC, as well as fewer in AD than NAD (not significantly) (Fig. 5c, g; Additional file 2: Table S3).

As in the discovery cohort, the corresponding size of distribution mode of GABRD^+^-carrying pTau217 and GPR162^+^-carrying pTau217 EVs were significantly smaller in AD vs NAD in the validation cohort (Fig. 5d, h). ROC analysis was performed to evaluate differential diagnosis performance. An integrative model combining age, the ratio of GABRD^+^-carrying pTau217 EVs and GPR162^+^-carrying pTau217 EVs, the corresponding size of distribution mode of GABRD^+^-carrying pTau217 EVs and GPR162^+^-carrying pTau217 EVs effectively discriminated AD from HC with an AUC of 0.93 (95% CI 0.879–0.977; sensitivity = 91.67%, specificity = 82.61%) (Fig. 5i) and distinguish AD from NAD with an AUC of 0.90 (95% CI 0.815–0.965; sensitivity = 89.58%, specificity = 75.86% (Fig. 5j).

Given the nature of multiple features analyzed, we conducted additional analyses (Additional file 2: Table S5) to evaluate the impact weights of each factor on disease discrimination. In discover cohort, as expected, GABRD^+^ carrying pTau217 EVs (*p* < 0.0001), GABRD^+^ EVs (*p* < 0.0001) and GPR162^+^ carrying pTau217 EVs (*p* < 0.05) were significantly associated with the diagnosis of AD vs HC. The remaining factors exhibited a comparatively lesser impact on the diagnoses of AD and HC when compared to these factors. Several factors, specifically GPR162^+^ carrying pTau217 EVs (*p* < 0.0001), GABRD^+^ carrying pTau217 EVs (*p* < 0.001), the distribution of GABRD^+^ carrying pTau217 EVs (*p* < 0.0001), the distribution of GPR162^+^ carrying pTau217 EVs (*p* < 0.0001), were significantly associated with the differential diagnosis of AD vs NAD. The impact of remaining factors on the differential diagnosis of AD and NAD was relatively weaker compared to the aforementioned factors. In validation cohort, GABRD^+^ carrying pTau217 EVs (*p* < 0.0001), the distribution of GABRD^+^ carrying pTau217 EVs (*p* < 0.0001), the distribution of GPR162^+^ carrying pTau217 EVs (*p* < 0.05) and GPR162^+^ carrying pTau217 EVs (*p* < 0.05) were significantly associated with the diagnosis of AD vs HC. And the factors, including pTau217^+^ EVs (*p* < 0.0001), the distribution of GABRD^+^ carrying pTau217 EVs (*p* < 0.0001), the distribution of GPR162^+^ carrying pTau217 EVs (*p* < 0.0001), GABRD^+^ carrying pTau217 EVs (*p* < 0.05) and GABRD^+^ EVs (*p* < 0.05) were significantly associated with the differential diagnosis of AD vs NAD. The influence of remaining factors on the diagnosis/differential diagnosis of AD was comparatively much weaker than those of the aforementioned factors.

In addition, we conducted DeLong comparisons to further test whether the integrated model has a significantly different AUC from single-factor diagnostic model. The findings revealed that, in comparing AD with HC in discovery cohort, the AUC was 0.9166 for GABRD^+^-carrying pTau217 EVs, 0.8183 for GPR162^+^-carrying pTau217 EVs, 0.5149 for the distribution of GABRD^+^ carrying pTau217 EVs, and 0.5211 for the distribution of GPR162^+^ carrying pTau217 EVs. The integrated model outperformed any single diagnostic model in differentiating AD from HC, exhibiting an AUC of 0.96. Furthermore, DeLong's test demonstrated that the integrated model significantly differed in AUC from each individual factor with *p* < 0.05, *p* < 0.01, *p* < 0.001, *p* < 0.001, respectively. Regarding AD vs NAD in discovery cohort, the AUC was 0.5079 for GABRD^+^-carrying pTau217 EVs, 0.7309 for GPR162^+^-carrying pTau217 EVs, 0.7422 for the distribution of GABRD^+^ carrying pTau217 EVs, and 0.6756 for the distribution of GPR162^+^ carrying pTau217 EVs. The integrated model performed even better in differentiating AD from NAD, with an AUC of 0.91. DeLong's test demonstrated that the integrated model significantly differed in AUC from each individual factor with *p* < 0.001, *p* < 0.001, *p* < 0.001, *p* < 0.001, respectively. In the validation cohort, consistent results were detected. In comparing AD with HC in validation cohort, the AUC was 0.7176 for GABRD^+^-carrying pTau217 EVs, 0.5452 for GPR162^+^-carrying pTau217 EVs, 0.5272 for the distribution of GABRD^+^ carrying pTau217 EVs, and 0.5108 for the distribution of GPR162^+^ carrying pTau217 EVs. The integrated model outperformed in differentiating AD from HC, with an AUC of 0.93. And we use DeLong’s test to demonstrate that the integrated model has a significantly different AUC from each individual factor with *p* < 0.001, *p* < 0.001, *p* < 0.001, *p* < 0.001, respectively. Concerning AD vs NAD in validation cohort, the AUC was 0.5875 for GABRD^+^-carrying pTau217 EVs, 0.5760 for GPR162^+^-carrying pTau217 EVs, 0.5329 for the distribution of GABRD^+^ carrying pTau217 EVs, and 0.5953 for the distribution of GPR162^+^ carrying pTau217 EVs. The integrated model performed even better in differentiating AD from NAD, with an AUC of 0.90. DeLong's test demonstrated that the integrated model significantly differed in AUC from each individual factor with *p* < 0.001, *p* < 0.001, *p* < 0.05, *p* < 0.05, respectively. This analytical result reinforces our claim that, while a single-factor model may exhibit a relatively limited discriminative capacity when comparing HC with AD or AD with NAD, the integrated model not only successfully distinguished between HC and AD but also effectively separated AD from NAD.

Finally, we performed bootstrapping analysis on the combined data of two large cohorts to compare between groups. The random resampling was conducted 1000 times, and the results are presented in Additional file 2: Table S6. The analysis focused on inter-group comparisons, and the results showed that compared to the HC group, both the AD and NAD groups showed significant statistical differences in GABRD^+^ carrying pTau217 EVs and GPR162^+^ carrying pTau217 EVs (*p* < 0.01). Additionally, compared to the HC group, the corresponding size of distribution mode of GABRD^+^ carrying pTau217 EVs and GPR162^+^ carrying pTau217 EVs differed significantly in both the AD and NAD groups (*p* < 0.05); and the difference was also significant between AD and NAD groups (*p* < 0.05).

## Discussion

In this study, we successfully identified two neuronal EVs markers, GABRD and GPR162, which exhibited enrichment in the cortex and hippocampus. These markers, in conjunction with the established AD-associated molecule pTau217, demonstrated significant diagnostic value not only in distinguishing AD from HC, but also in differentiating AD from NAD.

Blood-based diagnosis of CNS-specific EVs is challenging due to marker specificity and assay reproducibility. Previously described neuronal EVs, such as L1CAM- and NCAM-positive EVs, can be produced by other organ systems, especially in neoplastic diseases [7, 10, 11, 21, 45, 46]. One group has even questioned whether L1CAM is associated with EVs membrane in plasma [34]. To identify potential markers, we conducted extensive mass spectrometry on EVs from primary mouse cortical and hippocampal neuronal cultures (two regions predominantly involved in AD) and identified GABRD and GPR162 as candidate markers which were further validated using human brain tissue. Our study confirmed the primary expression of GABRD and GPR162 in neurons of human brain tissue sections in the cortex, hippocampus, cerebellum, and caudate nucleus, albeit with lower expression in the caudate nucleus and cerebellum (Fig. 1). This suggested that regionally enriched EVs carrying GABRD and GPR162 in plasma could potentially serve as more robust AD diagnostic markers considering the pathogenesis and onset regions of AD.

The free form of blood marker pTau217 has gained attention recently for its consistent increased in AD patients [4, 30, 35]. However, it remains unclear whether blood free form pTau217 is solely produced in the CNS and if it can distinguish between AD and NAD. Our study differs from previous reports in two ways: 1) alterations in pTau217 are the portion carried by neural-specific EVs, and 2) the numbers of EVs carrying pTau217 are decreased in AD patients compared to HC and NAD subjects (Figs. 4, 5). Further discussion is provided below.

To authenticate and establish the truly presence of GABRD, GPR162, and pTau217 on the surface of EVs, we conducted comprehensive validations from multiple perspectives, including cryo-EM, Western blot, NTA, NanoFCM (Figs. 2, 3) and STORM (Fig. 2). Three-dimensional visualization provided by the super-resolution microscopy technique unambiguously showed that GABRD/GPR162, pTau217, along with CD9, were on the same plasma EV surface (Fig. 2; Additional file 3: Video S1 and Additional file 4: Video S2). We demonstrated that GABRD and GPR162 were indeed surface markers associated with EVs and specific to brain regions. It is noteworthy that the particle size revealed through STORM imaging was larger in comparison to those observed through cryo-EM and NTA techniques (Fig. 2). This discrepancy can be attributed to two plausible factors. First, the STORM technique employs an analytical algorithm to reconstruct the acquired sequential frames prior to yielding the final image [57]. Thus, it should be emphasized that, unlike the well-established cryo-EM and NTA techniques, this algorithm-based approach may introduce certain bias when characterizing the size of EVs. Second, the process of fixation during sample preparation could have led to an increase in particle size. To state if differently, the data obtained through cryo-EM and NTA techniques (Fig. 2a, b) tend to provide a more representative depiction of the true size of particles.

Another notable technical advancement in this investigation is the application of a cutting-edge nanoscale flow cytometry (NanoFCM) equipment in a clinical study. In contrast to the Apogee technology that has been used by several groups [5, 19, 50, 59], NanoFCM could detect particles smaller than 80 nm with greater precision and efficiently overcame low reproducibility encountered during EVs enrichment [50, 51]. The sensitive detection range of NanoFCM has expanded the breadth of EVs sizes detection. Based on NanoFCM analyses, assays for GABRD^+^, GPR162^+^, and pTau217^+^ EVs had high accuracy and reproducibility (Fig. 3). Furthermore, NanoFCM not only detects the ratio of labeled EVs but also presents the corresponding size distribution of these specifically labeled EVs, offering novel research tools for the comprehensive characterization of EVs.

From a clinical diagnostic standpoint, the main findings of the current research focused on two major observations. First, the study showed that the proportion of GABRD^+^/GPR162^+^ EVs carrying pTau217 accurately distinguished AD patients from HC (AUC = 0.9166, GABRD^+^-carrying pTau217 EVs; 0.8183, GPR162^+^-carrying pTau217 EVs) (Additional file 2: Table S4). The diagnostic ROC achieved was close to what has been described for the performance of NMDAR2A [50]. However, to date, no EVs markers, including NMDAR2A reported recently by us (Additional file 2: Fig. S1), can differentiate AD from NAD readily. This challenge was met with our second major discovery, i.e., the alteration of the size distribution of EVs in AD was different from NAD patients. Specifically, we quantified the number of EVs with maximal positive frequency (i.e., the corresponding size distribution mode of positive EVs) as another evaluation index. By combining the ratio of GABRD^+^- or GPR162^+^- carrying pTau217 EVs, the corresponding size of distribution mode of GABRD^+^- or GPR162^+^- carrying pTau217 EVs, and the age of the subjects, the logistic regression model not only accurately discriminated between AD patients and HC (AUC = 0.96), but also effectively distinguished AD patients from NAD (AUC = 0.91). Cross-validation with an independent cohort confirmed that the combination of the ratio and the corresponding size of distribution mode of GABRD^+^- or GPR162^+^- carrying pTau217 EVs, and age can accurately discriminate between AD vs HC (AUC = 0.93), as well as AD vs NAD (AUC = 0.90). Of note, age as an independent variable (Additional file 2: Table S4) is not surprising, given that the most important risk factor in AD development is aging itself. Nevertheless, it needs to be stressed that it is the combination of these factors that can accurately discriminate between AD vs HC and AD vs NAD, further emphasizing on the complexity or heterogeneity of various dementias. Indeed, currently, there is no single fluid biomarker that can effectively differentiate different forms of dementias. It should also be noted that, with the nano-flowcytometry platform, alterations in the ratio and size of EVs carrying multiple markers can be detected in a single experiment, greatly improving biomarker assay efficiency.

Two additional facets of the biomarker results warrant further discussion. First, it is important to note that previous studies have found increased levels of EVs-associated proteins linked to AD development, including total tau, pTau, and Aβ, in plasma [9, 14, 24, 56]. These studies quantified the concentration of target proteins in neurogenic EVs after isolation from plasma by immunocapture and lysed before various measurements, which is quite different from the current study, where we quantify the ratio of numbers of EVs carrying different markers. The mechanism underling the observation of decreased neurogenic EVs in AD compared with HC remains to be further investigated. Several studies have shown that the development of AD is often accompanied by decreased levels of synaptic proteins, possibly reflecting characteristic synaptic dysfunction in neurodegeneration [1, 12, 13, 56]. In our previous studies, the proportion of NMDAR2A-carrying EVs detected in peripheral blood was also significantly reduced [50]. Therefore, the decrease in GABRD^+^ or GPR162^+^ carrying pTau217 EVs may simply be due to neuronal loss as dementia progresses, although other mechanisms require further investigation. The second point of discussion relates to the change in particle size in NAD vs. AD. Previous research has shown that EVs’ size, concentration, composition, and function vary with disease [54]. For instance, Lo TW et al. observed a reduction in the mean size of EVs in the serum of ALS patients. They posited that EV size might influence cargo or vice versa, given that ALS serum EVs, being smaller than control EVs, exhibited enrichment in upregulated miRNAs. However, the biological implications of EV size heterogeneity remain undisclosed in their study [27]. Another study suggested that the cell activation status and size of EVs can affect their membrane protein composition and functional capacity [38]. The difference in EVs size distribution mode between AD and NAD, shown in our study, could provide insight into dementia mechanisms. In future investigations, an experiment analyzing various-sized EVs from different dementias, e.g., with extensive profiling techniques, could be conducted.

It cannot be overemphasized that there are several limitations to the study. First, the updated diagnostic criteria for AD are heavily based on a framework to identify CSF Aβ (A), tau (T), and a neurodegeneration (N) marker, reflecting disease stage [22]. Others have tried to develop a similar scheme for a blood test. To this end, our study did not include Aβ, a biomarker of A, although pTau217 (T) and two neuronal markers (N) were tested. This is because, in a recent study, we had tested the ability of Aβ40 and Aβ42 associated with EVs to assist with diagnosis of AD, but the results were unsatisfactory (AUC 0.696–0.759). In future studies, the investigation will include EVs carrying Aβ40 and Aβ42, as well as explore EVs-related biomarkers for early AD diagnosis. Second, the study’s cohorts had differences in gender ratios and MMSE scores between AD and NAD groups, which could be due to the differences of patients enrolled from geographically vast different hospitals in China. Therefore, the main results must be validated in other independent cohorts, as well as the cohorts collected from less developed areas or countries in future clinical research. Third, although the average age of our cohorts was relatively young, the CSF testing and/or PET-CT affirmed the absence of an AD molecular signature in HC and NAD patients. However, the precise categorization of NAD subjects, such as distinguishing Lewy body dementia or frontotemporal dementia (with or without TDP-43 inclusions), was not feasible in the current investigation. Subclassifying the NADs stands as a key objective for our future research. Fourth, our cohorts also lack several important parameters, including corresponding magnetic resonance imaging (MRI) for everyone, and none of the cases had pathological confirmations—a challenging endeavor even in developed countries with decades of history in cohort studies. In future studies, in addition to independent validation using CSF/PET biomarkers, we intend to align molecular biomarkers, at the very least, with MRI data and integrate these diverse data types to provide a more comprehensive understanding of AD. Finally, in this study, our primary findings focus on the differential diagnosis of AD from other forms of dementia—an inherently significant issue. We recognize the merit in testing these novel markers among individuals with mild cognitive impairment (MCI). In the future, we plan to expand our studies to incorporate the intermediate or prodromal stage of AD. This will not only enhance the comprehensiveness of our findings but also contribute to the broader goal of early identification and intervention in AD.

In summary, this study provides compelling evidence that brain regionally enriched neurogenic EVs carrying pTau217, and their particle number and size distribution in plasma can be a reliable screening test for AD diagnosis and differential diagnosis. Furthermore, the biomarker results exhibit a high degree of consistency with PET imaging and CSF results. Compared to PET imaging and CSF analysis, two “gold standard” tests currently used for clinical confirmation of AD, this nanoflow cytometric-based assay is faster, less expensive, more convenient, and accessible in routine clinical practice. The assay would be even more powerful if it could detect dementia at earlier clinical stages of dementia, including in those with MCI. Moreover, the brain region-specific biomarker results provide new insights into the underlying mechanisms of AD and other dementias. Finally, a reliable blood test is essential to recruit patients and healthy controls for various clinical trials in the future.

### Supplementary Information

**Additional file 1.** Excel file contained mass spectrometry results of mouse cortical and hippocampal neurons EVs. **Table S1**: Mass spectrometry results of mouse cortical neurons EVs. **Table S2**: Mass spectrometry results of mouse hippocampal neurons EVs.

**Additional file 2. Table S3**: Biomarker levels of the discovery and validation cohorts. **Table S4**: Areas under the curve for ROC analyses. **Table S5**: The impact weights of each factor on AD discrimination. **Table S6**: The results of bootstrapping analysis. **Fig. S1**: The discriminatory efficacy of NMDAR2A and L1CAM in distinguishing AD from NAD. **Fig. S2**: Study design and procedures. **Fig. S3**: Labeling stability of NLGN3. **Fig. S4**: The expression of GABRD/GPR162 in a subset of neurons. **Fig. S5**: The full length of the gel for the proteins of Western blot in Fig. 2c. **Fig. S6**: The pattern of particle size variation.

**Additional file 3. Video S1:** Three-dimensional view of co-localization of GABRD, CD9 and pTau217.

**Additional file 4. Video S2**: Three-dimensional view of co-localization of GPR162, CD9 and pTau217.

## Data Availability

The datasets supporting the conclusions of this article are included within the article and its additional files.

## Abbreviations

AD: Alzheimer’s disease
EVs: Extracellular vesicles
GABRD: Gamma-aminobutyric acid receptor delta subunit
GPR162: G protein-coupled receptor 162
pTau217: Tau phosphorylated at Thr217
CNS: Central nervous system
MCI: Mild cognitive impairment
HC: Healthy control
NAD: Non-AD dementia
Aβ: Amyloid β
DLB: Dementia with Lewy body
CSF: Cerebrospinal fluids
NfL: Neurofilament light chain
Aβ40: Amyloid beta 1–40
Aβ42: Amyloid beta 1–42
NDEs: Neural-derived exosomes
NMDAR2A: *N*-Methyl-d-aspartate receptor 2A
L1CAM: L1 cell adhesion molecule
NLGN3: Neuroligin 3
CV: Coefficients of variation
PBS: Phosphate buffered saline
IHC: Immunohistochemical
Cryo-EM: Cryo-electron microscopy
NTA: Nanoparticle Tracking Analysis
STORM: Stochastic optical reconstruction microscopy
IgG: Immunoglobulin G
PFA: Paraformaldehyde
PFS: Perfect Focus System
ROC: Receiver Operating Characteristic
AUC: Area under the curve
SD: Standard deviation
CI: Confidence interval
MMSE: Mini-mental State Examination
MoCA: Montreal Cognitive Assessment
t-tau: Total-tau
PIB: Pittsburgh compound B
PET: Positron Emission Tomography
HDL: High-density lipoprotein
LDL: Low-density lipoprotein
PSMs: Peptide-spectrum matches
FDR: False discovery rate
TBST: Tris Buffered Saline with Tween 20
MRI: Magnetic resonance imaging
