# Supplementary material for: Blood-based CNS regionally and neuronally enriched extracellular vesicles carrying pTau217 for Alzheimer’s disease diagnosis and differential diagnosis
Source: Acta Neuropathol Commun. 2024 Mar 5;12:38. doi: 10.1186/s40478-024-01727-w (PMC10913681; doi:10.1186/s40478-024-01727-w)
Supplement: Supplementary file 2 — Additional file 2. Table S3: Biomarker levels of the discovery and validation cohorts. Table S4: Areas under the curve for ROC analyses. Table S5: The impact weights of each factor on AD discrimination. Table S6: The results of bootstrapping analysis. Fig. S1: The discriminatory efficacy of NMDAR2A and L1CAM in distinguishing AD from NAD. Fig. S2: Study design and procedures. Fig. S3: Labeling stability of NLGN3. Fig. S4: The expression of GABRD/GPR162 in a subset of neurons. Fig. S5: The full length of the gel for the proteins of Western blot in Fig. 2c. Fig. S6: The pattern of particle size variation. [file 40478_2024_1727_MOESM2_ESM.docx]

**Supporting Information_Acta Neuropathologica Communications**

**Blood-Based CNS Regionally and Neuronally Enriched Extracellular Vesicles Carrying pTau217 for Alzheimer’s Disease Diagnosis and Differential Diagnosis**

Zhen Guo^1†^, Chen Tian^1†^, Yang Shi^1,2^, Xue-Ru Song^1^, Wei Yin^3^, Qing-Qing Tao^4^, Jie Liu^5^, Guo-Ping Peng^6^, Zhi-Ying Wu^4^, Yan-Jiang Wang^5^, Zhen-Xin Zhang^7^, Jing Zhang^1,8^*

†The authors contributed equally to this article.

* Corresponding Author: Jing Zhang, Department of Pathology, The First Affiliated Hospital, Zhejiang University School of Medicine, Zhejiang, Hangzhou, 310003 China. Email: [jzhang1989@zju.edu.cn](mailto:jzhang1989@zju.edu.cn);

**List of Supplementary Materials**

**Table S1** Mass spectrometry results of mouse cortical neurons EVs (in the Excel file)

**Table S2** Mass spectrometry results of mouse hippocampal neurons EVs (in the Excel file)

**Video 1** Three-dimensional view of co-localization of GABRD, CD9 and pTau217

**Video 2** Three-dimensional view of co-localization of GPR162, CD9 and pTau217

**Table S3** Biomarker levels of the discovery and validation cohorts

**Table S4** Areas under the curve for ROC analyses

**Table S5** The impact weights of each factor on AD discrimination

**Table S6** The results of bootstrapping analysis

**Fig. S1** The discriminatory efficacy of NMDAR2A and L1CAM in distinguishing AD from NAD

**Fig. S2** Study design and procedures

**Fig. S3** Labeling stability of NLGN3

**Fig. S4** The expression of GABRD/GPR162 in a subset of neurons

**Fig. S5** The full length of the gel for the proteins of Western blot in Fig. 2c

**Fig. S6** The pattern of particle size variation

This supplementary material has been provided by the authors to give readers additional information about their work.

**Table S3 Biomarker levels of the discovery and validation cohorts**

| **EVs markers (Lg (positive particle %) ± S.D.)** | | | | | | |
| --- | --- | --- | --- | --- | --- | --- |
| **Marker** | **Discovery cohort (n = 310)** | | | **Validation cohort (n = 213)** | | |
|  | **HC**  **(n = 85)** | **AD**  **(n = 132)** | **NAD**  **(n = 93)** | **HC**  **(n = 70)** | **AD**  **(n = 99)** | **NAD**  **(n = 44)** |
| GABRD | 0.6246±0.2328 | 0.4825±0.2767  *** | 0.2434±0.3611  **** | 0.5299±0.2437 | 0.2918±0.3053  **** | 0.3391±0.3541  ** |
| GPR162 | 0.6716±0.2201 | 0.5384±0.2219  **** | 0.6767±0.1675 | 0.4721±0.3006 | 0.4334±0.3046 | 0.5079±0.3401 |
| pTau217 | 0.8569±0.1833 | 0.3381±0.3726  **** | 0.5097±0.2546  **** | 0.6880±0.2525 | 0.5279±0.2626  ** | 0.5723±0.3353 |
| GABRD^+^ carrying pTau217 EVs | 0.4429±0.2013 | 0.04244±0.2407  **** | 0.03368±0.3056  **** | 0.3005±0.2387 | 0.06877±0.3235  **** | 0.1180±0.4435 |
| GPR162^+^ carrying pTau217 EVs | 0.2962±0.2928 | -0.08692±0.2951  **** | 0.1716±0.2677  * | 0.2207±0.3725 | 0.1491±0.3730  * | 0.2358±0.4035 |
| **Size distribution (nm ± S.D.)** | | | | | | |
| GABRD^+^ carrying pTau217 EVs | 170.2±30.27 | 166.9±24.19 | 187.8±38.25  **** | 162.5±21.25 | 162.0±19.25 | 166.4±17.97  ** |
| GPR162^+^ carrying pTau217 EVs | 165.4±25.38 | 164.8±13.80 | 173.1±15.26  *** | 176.9±34.34 | 171.2±25.65 | 175.3±29.61  * |

**p* <0.05, ***p* <0.01, ****p* <0.001, *****p* <0.0001. one-tailed nonparametric ANOVA followed by Kruskal-Wallis test.

Abbreviations: HC, healthy control; AD, Alzheimer’s disease; NAD, Non-AD dementia; S.D., Standard Deviation; GABRD, Gamma-aminobutyric acid receptor delta subunit; GPR162, G-protein coupled receptor 162; pTau217, tau phosphorylated at Thr217; EVs, extracellular vesicles.

**Table S4 Areas under the curve for ROC analyses**

| **Comparison** | **Marker** | **AUC** | **95% CI** | **Cut-off** | **AUC** | **95% CI** | **Cut-off** |
| --- | --- | --- | --- | --- | --- | --- | --- |
|  |  | **Discovery Cohort** | | | **Validation Cohort** | | |
| **AD vs HC** | GABRD | 0.6607 | 0.5872~0.7342 | 0.648 | 0.7381 | 0.6605~0.8157 | 0.484 |
|  | GPR162 | 0.6679 | 0.5871~0.7488 | 0.677 | 0.5288 | 0.4349~0.6227 | 0.842 |
|  | pTau217 | 0.8958 | 0.8546~0.9370 | 0.562 | 0.6511 | 0.5628~0.7394 | 0.829 |
|  | GABRD^+^ carrying pTau217 EVs | 0.9166 | 0.8740~0.9592 | 0.332 | 0.7176 | 0.6388~0.7963 | 0.312 |
|  | GPR162^+^ carrying pTau217 EVs | 0.8183 | 0.7546~0.8820 | 0.130 | 0.5452 | 0.4515~0.6388 | 0.658 |
|  | The distribution of GABRD^+^ carrying pTau217 EVs | 0.5149 | 0.4308~0.5991 | 176.1 | 0.5272 | 0.4343~0.6200 | 209.5 |
|  | The distribution of GPR162^+^ carrying pTau217 EVs | 0.5211 | 0.4297~0.6125 | 142.9 | 0.5108 | 0.4188~0.6028 | 208.3 |
|  | Age | 0.6051 | 0.4658~0.7444 | _ | 0.5421 | 0.4478~0.6363 | _ |
| **AD vs NAD** | GABRD | 0.6900 | 0.6172~0.7629 | 0.351 | 0.5666 | 0.4589~0.6744 | 0.439 |
|  | GPR162 | 0.6840 | 0.6120~0.7559 | 0.597 | 0.5855 | 0.4822~0.6888 | 0.371 |
|  | pTau217 | 0.6385 | 0.5644~0.7126 | 0.312 | 0.5761 | 0.4732~0.6790 | 0.505 |
|  | GABRD^+^ carrying pTau217 EVs | 0.5079 | 0.4218~0.5940 | 0.312 | 0.5875 | 0.4761~0.6988 | 0.332 |
|  | GPR162^+^ carrying pTau217 EVs | 0.7309 | 0.6622~0.7996 | 0.097 | 0.5760 | 0.4689~0.6830 | 0.243 |
|  | The distribution of GABRD^+^ carrying pTau217 EVs | 0.7422 | 0.6769~0.8074 | 160.5 | 0.5329 | 0.4279~0.6381 | 156.0 |
|  | The distribution of GPR162^+^ carrying pTau217 EVs | 0.6756 | 0.5890~0.7623 | 159.5 | 0.5953 | 0.4937~0.6969 | 154.0 |
|  | Age | 0.6246 | 0.4959~0.7543 | _ | 0.5373 | 0.4451~0.6295 | _ |

Abbreviations: HC, healthy control; AD, Alzheimer’s disease; NAD, Non-AD dementia; S.D., Standard Deviation; GABRD, Gamma-aminobutyric acid receptor delta subunit; GPR162, G-protein coupled receptor 162; pTau217, tau phosphorylated at Thr217; EVs, extracellular vesicles; AUC, areas under the curve; ROC, receiver operating characteristic; CI, confidence interval;

**Table S5 The impact weights of each factor on AD discrimination**

| **Comparison** | **Marker** | **Discovery Cohort**  **(*p* value)** | **Validation Cohort**  **(*p* value)** |
| --- | --- | --- | --- |
| **AD vs HC** | GABRD | <0.0001 | 0.201 |
|  | pTau217 | 0.716 | 0.172 |
|  | GABRD^+^ carrying pTau217 EVs | <0.0001 | <0.0001 |
|  | GPR162 | 0.604 | 0.664 |
|  | GPR162^+^ carrying pTau217 EVs | 0.002 | 0.037 |
|  | The distribution of GABRD^+^ carrying pTau217 EVs | 0.696 | <0.0001 |
|  | The distribution of GPR162^+^ carrying pTau217 EVs | 0.749 | 0.007 |
|  | Age | 0.336 | 0.630 |
| **AD vs NAD** | GABRD | 0.482 | 0.008 |
|  | pTau217 | 0.778 | <0.0001 |
|  | GABRD^+^ carrying pTau217 EVs | 0.001 | 0.012 |
|  | GPR162 | 0.622 | 0.329 |
|  | GPR162^+^ carrying pTau217 EVs | <0.0001 | 0.464 |
|  | The distribution of GABRD^+^ carrying pTau217 EVs | <0.0001 | <0.0001 |
|  | The distribution of GPR162^+^ carrying pTau217 EVs | <0.0001 | <0.0001 |
|  | Age | 0.216 | 0.611 |

Abbreviations: HC, healthy control; AD, Alzheimer’s disease; NAD, Non-AD dementia; GABRD, Gamma-aminobutyric acid receptor delta subunit; GPR162, G-protein coupled receptor 162; pTau217, tau phosphorylated at Thr217; EVs, extracellular vesicles.

**Table S6 The results of bootstrapping analysis**

| **Factor** | **Group** | **N** | **Range** | **Minimum** | **Maximum** | **Mean** | | **Std. Deviation** | **Variance** |
| --- | --- | --- | --- | --- | --- | --- | --- | --- | --- |
|  |  | **Statistic** | **Statistic** | **Statistic** | **Statistic** | **Statistic** | **Std. Error** | **Statistic** | **Statistic** |
| GABRD^+^ carrying pTau217 EVs | HC | 155 | 1.602 | -0.699 | 0.903 | 0.340 | 0.022 | 0.278 | 0.077 |
|  | AD | 231 | 1.484 | -0.699 | 0.785 | 0.082 | 0.019 | 0.289 | 0.084 |
|  | NAD | 137 | 1.792 | -1.000 | 0.792 | -0.001 | 0.034 | 0.397 | 0.158 |
| GPR162^+^ carrying pTau217 EVs | HC | 155 | 1.929 | -1.000 | 0.929 | 0.189 | 0.032 | 0.403 | 0.163 |
|  | AD | 230 | 2.072 | -1.000 | 1.072 | 0.061 | 0.025 | 0.377 | 0.142 |
|  | NAD | 137 | 1.954 | -1.000 | 0.954 | 0.157 | 0.030 | 0.353 | 0.124 |
| The distribution of GABRD^+^ carrying pTau217 EVs | HC | 153 | 149.000 | 127.750 | 276.750 | 173.234 | 2.606 | 32.231 | 1038.811 |
|  | AD | 219 | 151.000 | 134.000 | 285.000 | 168.614 | 1.677 | 24.812 | 615.637 |
|  | NAD | 126 | 268.750 | 138.250 | 407.000 | 183.514 | 3.200 | 35.914 | 1289.843 |
| The distribution of GPR162^+^ carrying pTau217 EVs | HC | 154 | 144.500 | 125.750 | 270.250 | 164.101 | 1.899 | 23.566 | 555.353 |
|  | AD | 189 | 121.500 | 126.250 | 247.750 | 159.911 | 1.310 | 18.007 | 324.257 |
|  | NAD | 119 | 125.250 | 137.750 | 263.000 | 170.622 | 1.518 | 16.560 | 274.227 |

Abbreviations: HC, healthy control; AD, Alzheimer’s disease; NAD, Non-AD dementia; GABRD, Gamma-aminobutyric acid receptor delta subunit; GPR162, G-protein coupled receptor 162; pTau217, tau phosphorylated at Thr217; EVs, extracellular vesicles.

**
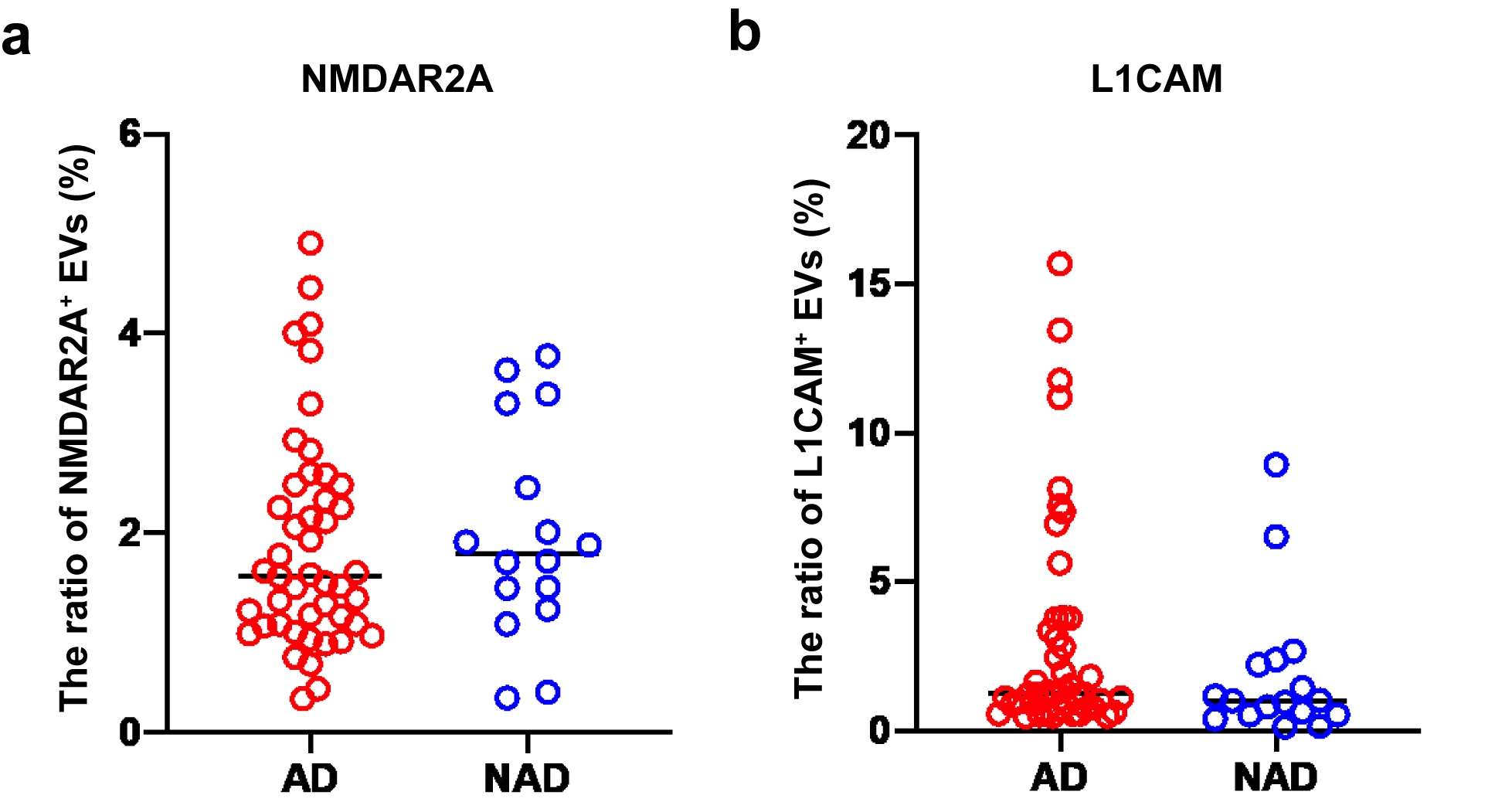
**

**Fig. S1 The discriminatory efficacy of NMDAR2A and L1CAM in distinguishing AD from NAD** (**a**) The ratio of NMDAR2A^+^ EVs in AD and NAD group (*p* = 0.604). (**b**) The ratio of L1CAM^+^ EVs in AD and NAD group (*p* = 0.128). The Y-axis signifies the ratio of EVs harboring the respective protein relative to the overall count of EVs.


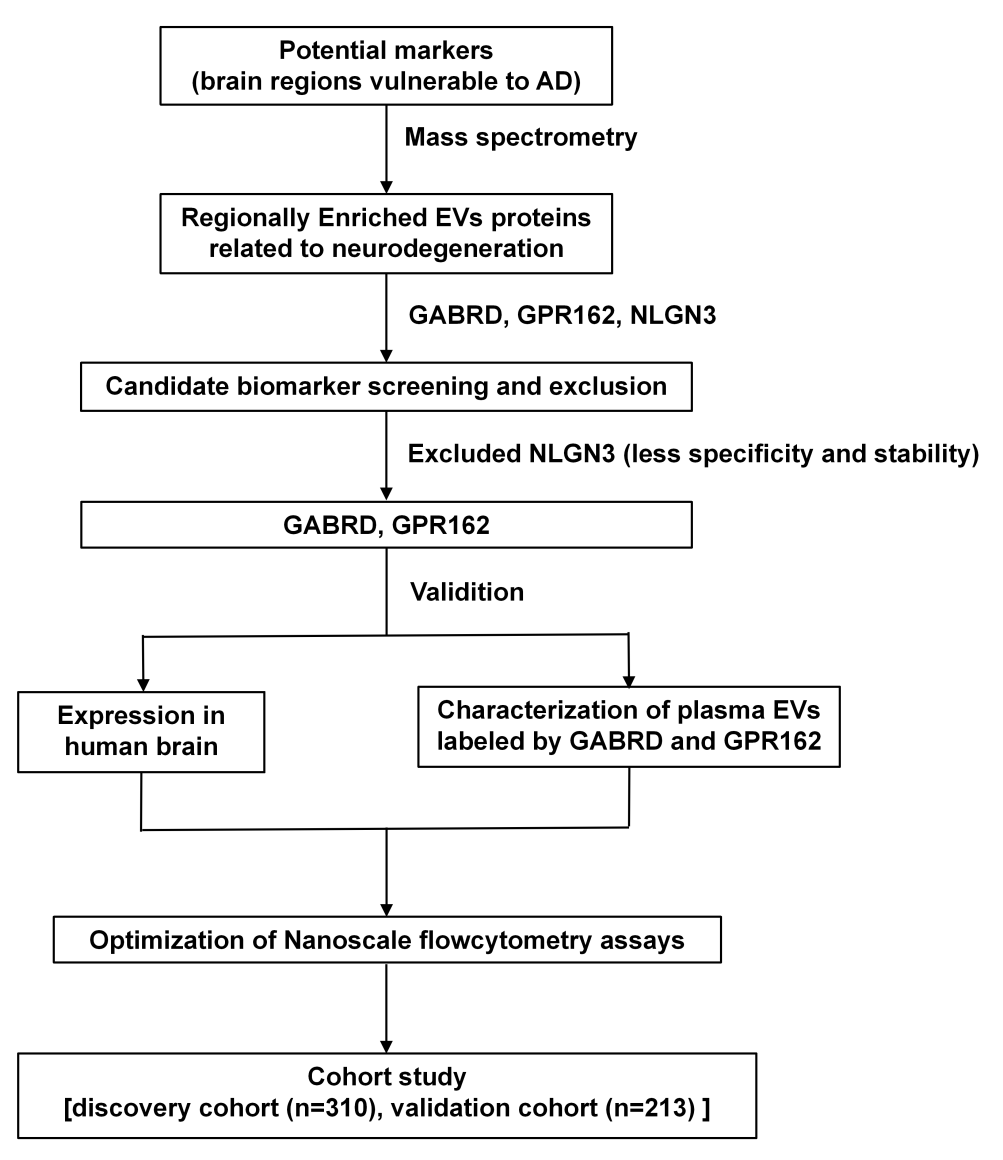


**Fig. S2 Study design and procedures.**

**
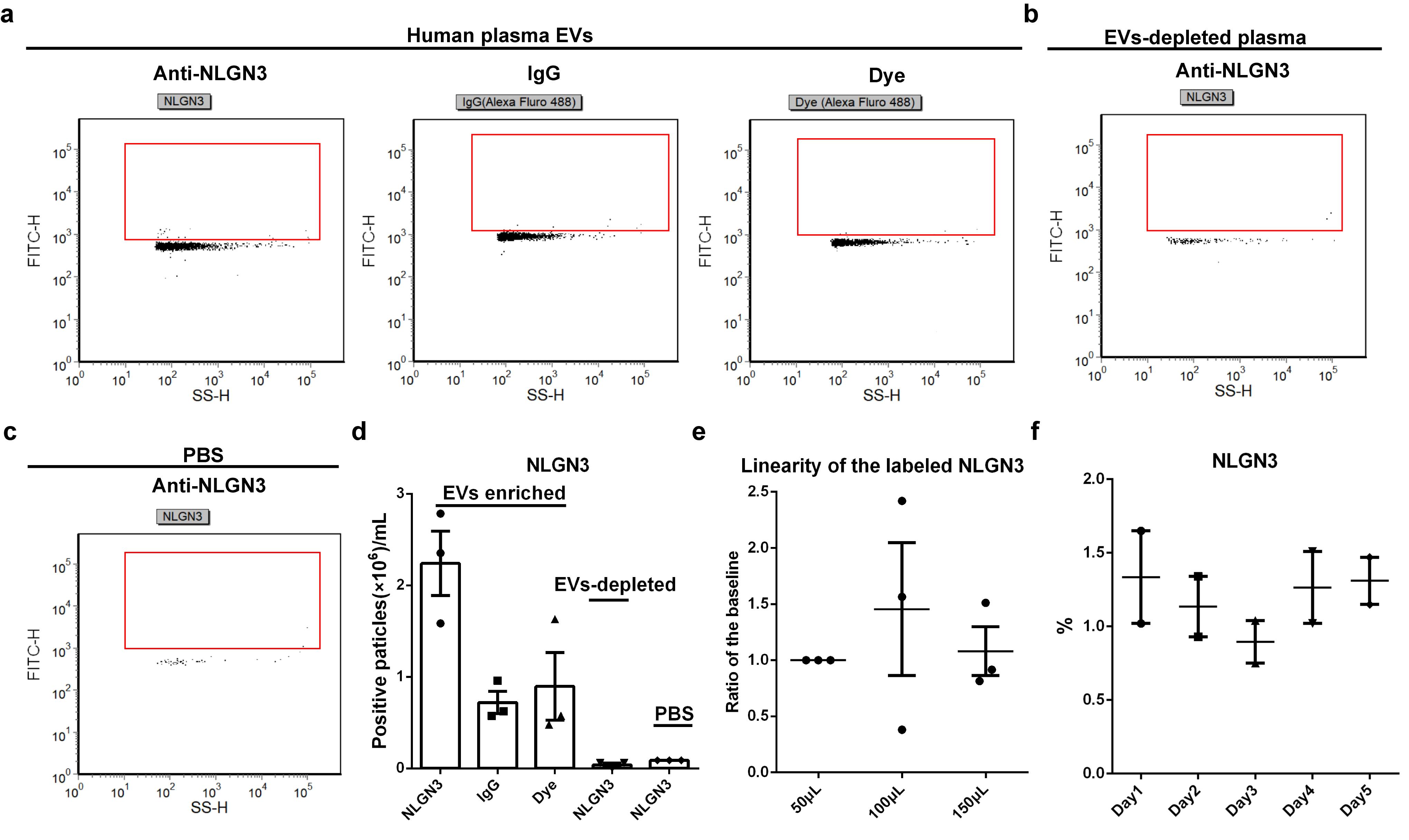
**

**Fig. S3 Labeling stability of NLGN3** (**a**) Example histograms showing populations of EVs which were positive for NLGN3 after labeling with fluorophore-conjugated antibody; plasma EVs labeled using fluorophore-conjugated immunoglobulin G isotype control for the indicated marker target antibody; and plasma EVs incubated with dye (fluorophore only, no antibody) control experiment. (**b**) Histogram of remaining particles after depletion of EVs from plasma by ultracentrifugation. (**c**) Histogram of PBS incubated with fluorophore-conjugated antibody. (**d-f**) Summary data from experiments demonstrating specificity of EVs assays (n = 3) (**d**), linearity in different dilutions of EVs plasma samples (n = 3) (**e**), and stability of reference plasma (two replicates run each day on 5 separate days of the experiment) for NLGN3 (**f**). Positive particles were circled out using red boxes.


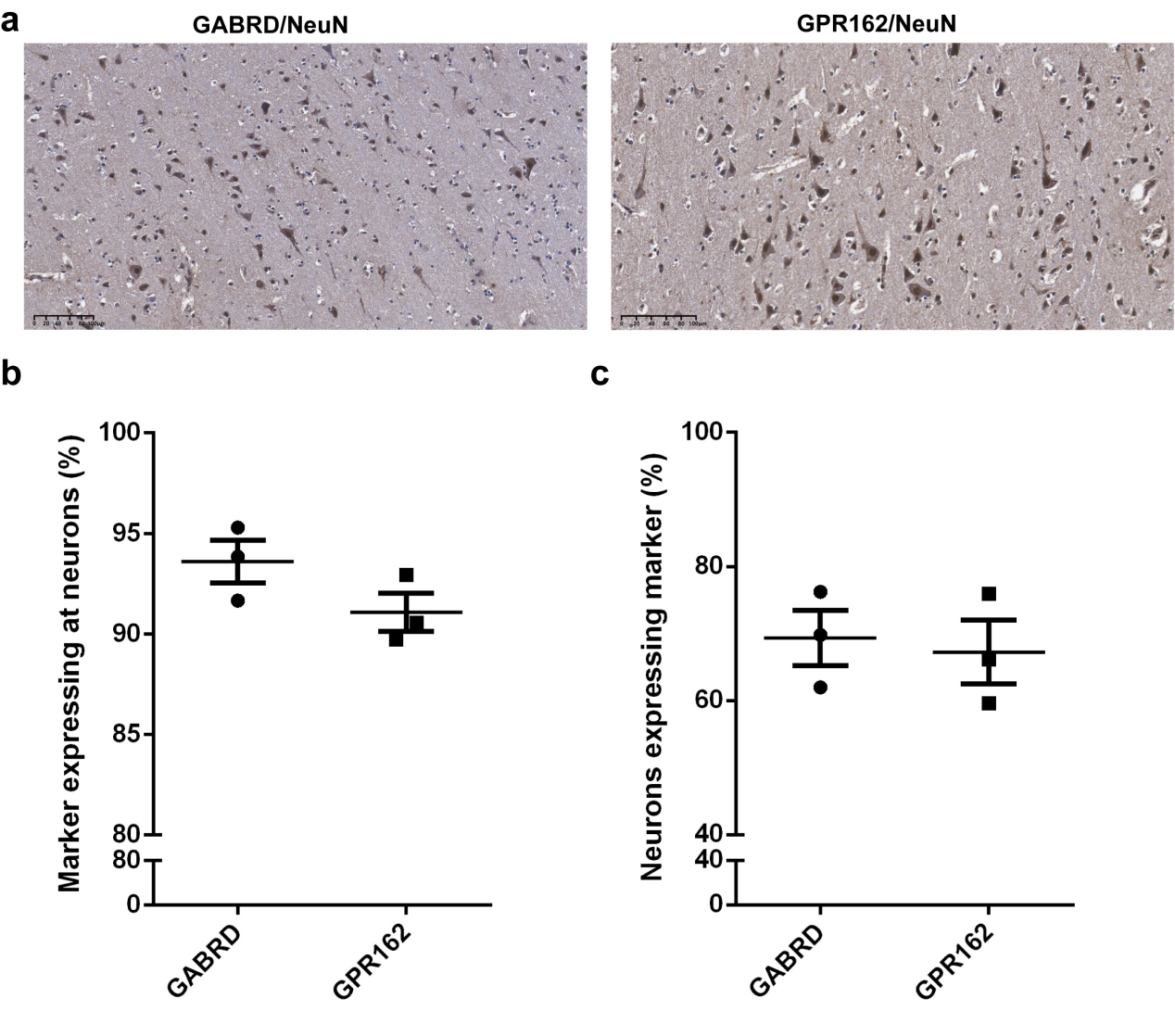


**Fig. S4 The expression of GABRD/GPR162 in a subset of neurons** (**a**) IHC analysis showed the co-stain of GABRD/GPR162 with neuronal marker NeuN in the cortex. (**b**) Statistics of GABRD/GPR162 expression in neurons. Neuronal cells expressing GABRD 93.60%±1.826% were neurons; Neuronal cells expressing GPR162 91.08%±1.660% were neurons (n = 3 subjects). (**c**) Statistics of neurons expressing GABRD/GPR162. 69.35%±7.151% neurons expressed GABRD; 67.28%±8.204% neurons expressed GPR162 (n = 3 subjects). Scale bar = 100 μm.


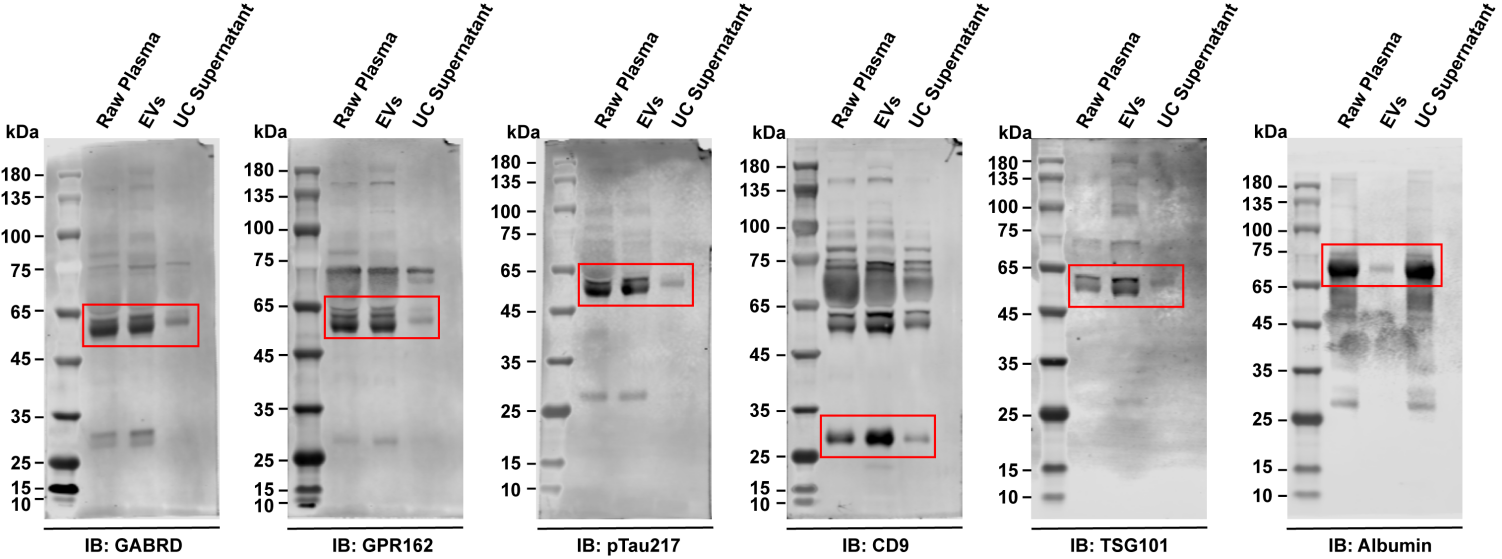


**Fig. S5 The full length of the gel for the proteins of Western blot in Fig. 2c**. The target bands in the Western blot were circled out using red boxes.


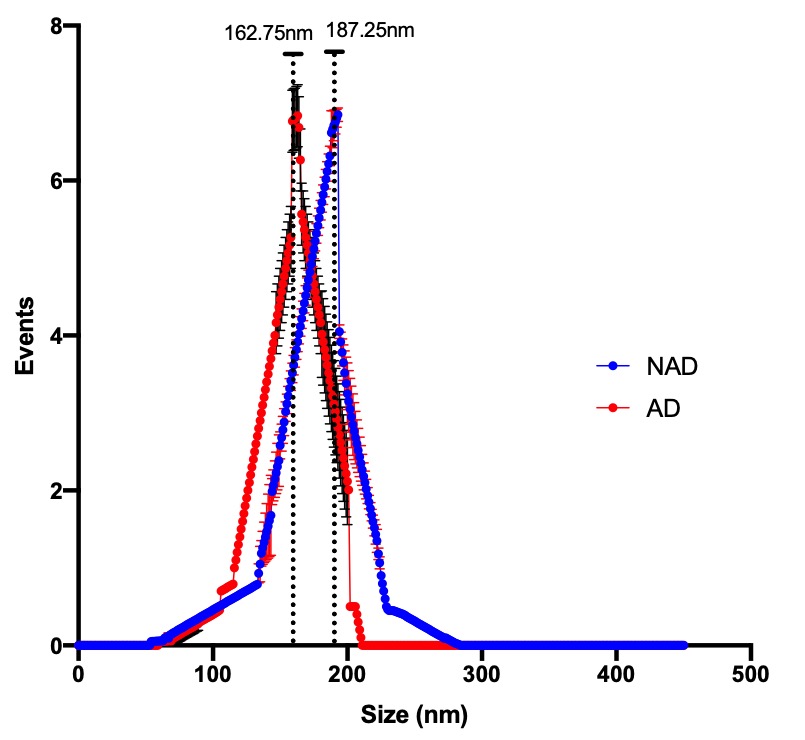


**Fig. S6 The pattern of particle size variation.** The pattern diagram depicted the particle size variation of “the corresponding size of the distribution mode of GABRD^+^- or GPR162^+^- carrying pTau217 EVs”. AD case (red), NAD case (blue) (n = 3).
